# S-Nitroso-L-Cysteine Stereoselectively Blunts the Deleterious Effects of Fentanyl on Breathing While Augmenting Antinociception in Freely-Moving Rats

**DOI:** 10.3389/fphar.2022.892307

**Published:** 2022-05-26

**Authors:** Paulina M. Getsy, Santhosh M. Baby, Ryan B. Gruber, Benjamin Gaston, Tristan H. J. Lewis, Alan Grossfield, James M. Seckler, Yee-Hsee Hsieh, James N. Bates, Stephen J. Lewis

**Affiliations:** ^1^ Department of Pediatrics, Case Western Reserve University, Cleveland, OH, United States; ^2^ Galleon Pharmaceuticals, Inc., Horsham, PA, United States; ^3^ Herman B Wells Center for Pediatric Research, Indiana University School of Medicine, Indianapolis, IN, United States; ^4^ Department of Biochemistry and Biophysics, University of Rochester Medical Center, Rochester, NY, United States; ^5^ Department of Biomedical Engineering, Case Western Reserve University, Cleveland, OH, United States; ^6^ Division of Pulmonary, Critical Care and Sleep Medicine, Case Western Reserve University, Cleveland, OH, United States; ^7^ Department of Anesthesia, University of Iowa, Iowa City, IA, United States; ^8^ Department of Pharmacology, Case Western Reserve University, Cleveland, OH, United States; ^9^ Functional Electrical Stimulation Center, Case Western Reserve University, Cleveland, OH, United States

**Keywords:** S-nitrosothiol, fentanyl, frequency of breathing, tidal volume, minute ventilation, noneupneic breathing index, Sprague Dawley rats

## Abstract

Endogenous and exogenously administered S-nitrosothiols modulate the activities of central and peripheral systems that control breathing. We have unpublished data showing that the deleterious effects of morphine on arterial blood-gas chemistry (i.e., pH, pCO_2_, pO_2_, and sO_2_) and Alveolar-arterial gradient (i.e., index of gas exchange) were markedly diminished in anesthetized Sprague Dawley rats that received a continuous intravenous infusion of the endogenous S-nitrosothiol, S-nitroso-L-cysteine. The present study extends these findings by showing that unanesthetized adult male Sprague Dawley rats receiving an intravenous infusion of S-nitroso-L-cysteine (100 or 200 nmol/kg/min) markedly diminished the ability of intravenous injections of the potent synthetic opioid, fentanyl (10, 25, and 50 μg/kg), to depress the frequency of breathing, tidal volume, and minute ventilation. Our study also found that the ability of intravenously injected fentanyl (10, 25, and 50 μg/kg) to disturb eupneic breathing, which was measured as a marked increase of the non-eupneic breathing index, was substantially reduced in unanesthetized rats receiving intravenous infusions of S-nitroso-L-cysteine (100 or 200 nmol/kg/min). In contrast, the deleterious effects of fentanyl (10, 25, and 50 μg/kg) on frequency of breathing, tidal volume, minute ventilation and non-eupneic breathing index were fully expressed in rats receiving continuous infusions (200 nmol/kg/min) of the parent amino acid, L-cysteine, or the D-isomer, namely, S-nitroso-D-cysteine. In addition, the antinociceptive actions of the above doses of fentanyl as monitored by the tail-flick latency assay, were enhanced by S-nitroso-L-cysteine, but not L-cysteine or S-nitroso-D-cysteine. Taken together, these findings add to existing knowledge that S-nitroso-L-cysteine stereoselectively modulates the detrimental effects of opioids on breathing, and opens the door for mechanistic studies designed to establish whether the pharmacological actions of S-nitroso-L-cysteine involve signaling processes that include 1) the activation of plasma membrane ion channels and receptors, 2) selective intracellular entry of S-nitroso-L-cysteine, and/or 3) S-nitrosylation events. Whether alterations in the bioavailability and bioactivity of endogenous S-nitroso-L-cysteine is a key factor in determining the potency/efficacy of fentanyl on breathing is an intriguing question.

## Introduction

Endogenous S-nitrosothiols (SNOs) regulate a variety of neural systems within the central (Lei et al., 1992; Lipton et al., 1993; Lipton et al., 1994; Takahashi et al., 2007; Tegeder et al., 2011; Raju et al., 2015; Tarasenko, 2015; Nakamura and Lipton, 2016) and peripheral nervous systems (Meller et al., 1990; Matsuda et al., 1995; Savidge, 2011; Lee et al., 2013; Tooker and Vigh, 2015; Gaston et al., 2020). SNOs exert their effects by mechanisms involving breakdown to nitric oxide (NO) followed by formation of dinitrosothiol-iron complexes that activate intracellular soluble guanylate cyclase and protein kinase G cell signaling (Mellion et al., 1983; Travis et al., 1996; Severina et al., 2003; Lima et al., 2010; Martínez-Ruiz et al., 2013; Marozkina and Gaston, 2015; Vanin, 2019), and by S-nitrosylation (transfer of NO^+^) of sulfur atoms within functional proteins (Jaffrey et al., 2001; Joksovik et al., 2007; Foster et al., 2009; Lima et al., 2010; Rudkouskaya et al., 2010; Marozkina and Gaston, 2012; Anand et al., 2014; Pires da Silva et al., 2016; Wynia-Smith and Smith, 2017; Stomberski et al., 2019; Marozkina and Gaston, 2020). S-nitroso-L-cysteine (L-CSNO) is an endogenous endothelium-derived SNO (Myers et al., 1990; Bates et al., 1991; Kukreja et al., 1993) that is synthesized and stored in vesicles of vascular endothelial cells (Seckler et al., 2020). L-CSNO may be actively/exocytotically released from the endothelium of peripheral vascular beds (Davisson et al., 1996a; Batenburg et al., 2004a; Batenburg et al., 2004b; Hashmi-Hill et al., 2007; Batenburg et al., 2009), and from neurogenic vasodilator nerves in peripheral vascular beds (Davisson et al., 1994; Davisson et al., 1996a; Davisson et al., 1996b; Davisson et al., 1997a; Possas and Lewis 1997; Possas et al., 2006).

Many of the pharmacological actions of SNOs such as L-CSNO and S-nitroso-β,β-dimethyl-L-cysteine, depend upon their stereoisomeric configuration (Davisson et al., 1996d; Lewis et al., 1996; Travis et al., 1996; Davisson et al., 1997b; Ohta et al., 1997; Travis et al., 1997; Hoque et al., 1999; Hoque et al., 2000; Travis et al., 2000; Lipton et al., 2001; Lewis et al., 2005a; Lewis et al., 2005b; Lewis et al., 2006a; Gaston et al., 2020). For instance, microinjections of L-CSNO into the nucleus tractus solitarius (NTS) lower arterial blood pressure in anesthetized rats (Ohta et al., 1997), and injections of L-CSNO into the lateral (Davisson et al., 1997b) or fourth (Lewis et al., 1996) ventricles of unanesthetized freely-moving rats elicit pronounced hemodynamic responses, however, injections of the D-isomer (D-CSNO) elicit minor responses. Potential stereoselective L-CSNO binding/recognition sites have not been fully identified, nevertheless we reported that L-CSNO, but not D-CSNO, directly activates voltage-gated K^+^-channels in a process that does not require the S-nitrosylation of the functional protein (Gaston et al., 2020). With respect to control of breathing, microinjections of L-CSNO into the NTS elevate minute ventilation (V_E_) in unanesthetized freely-moving rats (Lipton et al., 2001) by stereospecific mechanisms that are not related to L-CSNO decomposition to NO. In addition, it is evident that arterial injections of L-CSNO increases V_E_ in unanesthetized rats by activation of stereoselective processes in the carotid bodies, since 1) the L-CSNO-induced ventilatory responses were substantially diminished in rats that previously underwent bilateral transection of the carotid sinus nerves (CSNs) and 2) the L-CSNO-induced responses were not produced by D-CSNO (Gaston et al., 2020).

The involvement of nitrosyl factors, such as NO and SNOs, in the pharmacological actions of opioids has received considerable attention. For example, there is substantial evidence for nitrosyl factors playing roles in 1) opioid receptor (OR) signaling processes (Pol, 2007; Toda et al., 2009a; Toda et al., 2009b; Rodríguez-Muñoz and Garzón, 2013), and 2) opioid effects on a) vascular function and reactivity (Sahin et al., 2005; Kaye et al., 2006), b) pain processing (Pelligrino, et al., 1996; Maegawa and Tonussi, 2003; Cury et al., 2011; Hervera et al., 2011; Mehanna et al., 2018; Ortiz et al., 2020), c) vision (Someya et al., 2017), and d) inflammatory-immunoregulatory processes (Bilfinger, et al., 1998; Jan et al., 2011). Additionally, nitrosyl factors are involved in opioid-induced catalepsy (Erkent et al., 2006), tolerance to opioids (Kissin et al., 2000; Ozdemir et al., 2011; Durmus et al., 2014), and fentanyl pre-conditioning (Lu et al., 2014). Nonetheless, only a few studies have sought evidence as to potential roles for nitrosyl factors in the ventilatory depressant effects of opioids. For instance, the ventilatory depressant responses elicited by fourth ventricular infusions of morphine in awake dogs were reduced by prior injection of the NO synthase (NOS) inhibitor, L-nitro-arginine (L-NA), whereas injection of L-NA after the injection of morphine was ineffective (Pellegrino et al., 1996). Another study found that the ventilatory depressant effects of morphine in anesthetized cats are independent of neuronal NOS (nNOS) (Teppema et al., 2000). In addition, recent studies (Seckler et al., 2022) demonstrated that the ventilatory depressant effects of fentanyl were augmented in unanesthetized rats that had received the NOS inhibitor, N^G^-nitro-L-arginine methyl ester (L-NAME), suggesting that the dominant role for nitrosyl factors is to ameliorate the deleterious effects of opioids on ventilation. Although the mechanisms by which nitrosyl factors exert these effects are not clear, it is known that SNOs modulate G protein-coupled receptor signaling in a receptor-specific and reversible manner (Whalen et al., 1999; Kokkola et al., 2005; Nozik-Grayck et al., 2006; Whalen et al., 2007), and importantly, in the context of the present study, that neither SNO-L-CYS or S-nitroso-L-glutathione directly interact with μ-ORs (Kokkola et al., 2005).

Recently, we determined that the detrimental effects of morphine on arterial blood-gas chemistry (i.e., pH, pCO_2_, pO_2_, and SO_2_), Alveolar-arterial gradient (i.e., index of alveolar gas-exchange), respiratory frequency (f_R_), tidal volume (V_T_), and minute ventilation (V_E_) were markedly reduced in anesthetized rats receiving a continuous intravenous infusion of L-CSNO, but not those receiving D-CSNO infusion. Moreover, the antinociceptive effects of morphine were not reduced in the rats receiving either L-CSNO or D-CSNO. While furthering our knowledge about the roles of nitrosyl factors in ventilatory control processes (Stamler et al., 1997; Lipton et al., 2001; Gaston et al., 2006; Haldar and Stamler, 2013; Palmer et al., 2013a; Gaston et al., 2014; Palmer et al., 2015; Gaston et al., 2020; Getsy et al., 2021; Seckler et al., 2022) and establishing that L-CSNO has intriguing properties directly related to modulation of OR signaling cascades, the presence of anesthesia could be viewed as problematic, and the choice of morphine does not directly address the most urgent need to develop therapies against fentanyl, an analogue driving the current opioid crisis (Han et al., 2019; Torralva and Janowsky, 2019; Lutfy, 2020). As such, the major objective of the present study was to determine whether continuous intravenous infusions of L-CSNO at 100 or 200 nmol/kg/min could modulate the problematic effects of fentanyl given by consecutive injections of 10, 25, and 50 μg/kg, IV, on ventilatory parameters, f_R_, V_T_, and V_E_, and ventilatory stability, as defined by the non-eupneic breathing index (NEBI) (Getsy et al., 2014), in unanesthetized and unrestrained Sprague Dawley rats. A secondary objective was to determine whether infusion of L-CSNO modulates the antinociceptive actions of fentanyl in unanesthetized rats as determined by the radiant-heat tail-flick latency (TFL) assay (Henderson et al., 2014; Gaston et al., 2021). Finally, we report surprising, but intriguing, findings related to the ability of the peripherally restricted OR antagonist, naloxone methiodide (NLXmi) (Lewanowitsch and Irvine, 2002; Lewanowitsch, et al., 2006; Henderson et al., 2014), to markedly increase f_R_ in rats that had received prior injections of fentanyl, but not vehicle.

## Materials and Methods

### Permissions and Rats and Surgeries

All animal studies were carried out in accordance with the National Institutes of Health Guide for the Care and Use of Laboratory Animals (NIH Publication No. 80.23) revised in 1996. The protocols were approved by the Institutional Animal Care and Use Committees of the University of Virginia, Case Western Reserve University, and Galleon Pharmaceuticals, Inc. Adult male Sprague Dawley rats were obtained from Harlan Laboratories, Inc. (Indianapolis, IN, United States). These rats were caged in standard housing conditions in our vivaria with free access to food and water. Room temperature (22°C), humidity (48%–50%) and light-dark cycle (12:12 h) were maintained consistently in each vivarium and laboratory where the studies were performed. All protocols involved the use of rats that had been implanted with two intravenous jugular catheters exteriorized to the back of the neck as detailed previously (Davisson et al., 1996a; Davisson et al., 1996b; Davisson et al., 1996c; Davisson et al., 1996d; Davisson et al., 1997a). The jugular vein catheters were implanted under 2.5%–3.5% isoflurane anesthesia 7 days previously to be sure that the rats were free from surgical discomfort. On the day of the experiment, one catheter (PE-50 connected to PE-10, the PE-10 tubing inserted into the right jugular vein) allowed for the continuous intravenous infusion of vehicle (20 μl/min), L-CSNO (100 or 200 mol/kg/min), L-cysteine (200 nmol/kg/min) or D-CSNO (200 nmol/kg/min). The second catheter was inserted into the left jugular vein for the bolus injection of vehicle, fentanyl or NLXmi. It is important to note the infusions were maintained throughout the experiment.

### Whole Body Plethysmography Protocols

Ventilatory parameters (i.e., f_R_, V_T_, V_E_, and NEBI) were recorded in freely-moving rats by whole body plethysmography (PLY3223; Data Sciences International, St. Paul, MN, United States) as detailed previously (Getsy et al., 2020; Gaston et al., 2021; Seckler et al., 2022). The rats were given 60 min to acclimate to the chambers, thus allowing true resting (i.e., baseline) ventilatory parameters to be established. A description of the protocols undergone by the six treatment groups of rats and their average body weights are provided in Table 1. Briefly, treatment group one (i.e., Vehicle 1) rats received a continuous infusion of phosphate-buffered saline (PBS) (i.e., vehicle) at pH 7.2. After 45 min these rats received three injections of vehicle, each given 30 min apart. Thirty minutes after the third vehicle injection, the rats received an injection of NLXmi (2.5 mg/kg, IV). Treatment group two (i.e., Vehicle 2) rats received a continuous infusion of vehicle and after 45 min, injections of fentanyl at doses 10, 25, and 50 μg/kg, each given 30 min apart. Thirty minutes after injection of the 50 μg/kg dose of fentanyl, the rats received an injection of vehicle (100 μl/kg, IV). Treatment group three (i.e., L-CSNO) rats received a continuous infusion of L-CSNO (200 nmol/kg/min) and after 45 min, injections of fentanyl at doses 10, 25, and 50 μg/kg, each given 30 min apart. Thirty minutes after injection of a 50 μg/kg dose of fentanyl, the rats received an injection of NLXmi (2.5 mg/kg, IV). Treatment group four (i.e., Vehicle 3) rats received the same protocol as Vehicle 1 rats and served as the controls for treatment group five (i.e., L-Cysteine) and treatment group six (i.e., D-CSNO). L-Cysteine and D-CSNO rats received an infusion of L-cysteine (200 nmol/kg/min, IV) or D-CSNO (200 nmol/kg/min, IV) respectively and after 45 min, injections of fentanyl (10, 25, and 50 μg/kg) each given 30 min apart. Thirty minutes after the injection of the 50 μg/kg dose of fentanyl, the rats received an injection of NLXmi (2.5 mg/kg, IV). It is important to note that the infusions of the test agents were continued throughout the entire protocol (i.e., until 15 min after the injection of NLXmi or vehicle).

**TABLE 1:** Description of the six treatment groups used in the whole body plethysmography experiments.

Group	Weight (g)	Infusion	Bolus injections			Vehicle/NLXmi
Vehicle 1	317 ± 3	20 μl/min	Vehicle	Vehicle	Vehicle	NLXmi, 2.5 mg/kg
Vehicle 2	320 ± 3	20 μl/min	F10	F25	F50	Vehicle, 100 μL/kg
L-CSNO	319 ± 3	200 nmol/kg/min	F10	F25	F50	NLXmi, 2.5 mg/kg
Vehicle 3	320 ± 2	20 μl/min	Vehicle	Vehicle	Vehicle	NLXmi, 2.5 mg/kg
L-Cysteine	322 ± 3	200 nmol/kg/min	F10	F25	F50	NLXmi, 2.5 mg/kg
D-CSNO	318 ± 3	200 nmol/kg/min	F10	F25	F50	NLXmi, 2.5 mg/kg

L-CSNO, S-nitroso-L-cysteine; D-CSNO, S-nitroso-D-cysteine; NLXmi, naloxone methiodide. F10, F25, and F50 represent intravenous injections of 10, 25, or 50 μg/kg doses of fentanyl, respectively. There were 8 rats in each group. The body weights of the six groups of rats (mean ± SEM) were similar to one another (*p* > 0.05, for all comparisons).

Due to the closeness of the body weights of the six treatment groups of rats, ventilatory data, specifically V_T_ and V_E_, are presented without body weight corrections. Provided software (Fine Pointe, BUXCO) constantly corrected digitized values for changes in chamber temperature and humidity. Pressure changes associated with the respiratory waveforms were then converted to volumes (i.e., V_T_) using the algorithm of Epstein and colleagues (Epstein and Epstein, 1978; Epstein et al., 1980). Factoring in chamber temperature and humidity, the cycle analyzers filtered acquired signals, and Fine Pointe algorithms generated an array of box flow data that identified a waveform segment as an acceptable (eupneic) breath. From that data vector, the minimum and maximum values were determined. Flows at this point were considered to be box flow signals. From this array, minimum and maximum box flow values were determined and multiplied by a compensation factor provided by the selected algorithm (Epstein and Epstein, 1978; Epstein et al., 1980), thus producing V_T_ values that were used to determine the non-eupneic breathing events expressed as the non-eupneic breathing index (NEBI), reported as the percentage of non-eupneic breathing events per epoch (Getsy et al., 2014). All directly recorded parameters, including NEBI, were then extracted from the raw waveforms using Data Sciences International (St. Paul, MN, United States) proprietary Biosystem XA software (version 2.9.0.2) and proprietary FinePointe software (version v2.8.0), as described previously (Getsy et al., 2020; Gaston et al., 2021; Seckler et al., 2022) and as detailed in the Data Sciences International/Buxco website reference to the list of parameters provided by proprietary FinePointe Software using whole body plethysmography (https://www.datasci.com/products/buxco-respiratory-products/finepointe-whole-body-plethysmography). The BioSystem XA software extracts the waveforms that are analyzed by the FinePointe software that uses National Instruments Measurement Studio to perform the analyses (http://zone.ni.com/reference/en-XX/help/37263 6F-01/mstudiowebhelp/html/5d5b3031/).

### Antinociception Protocols

Antinociception status in unanesthetized rats was determined by a radiant heat tail-flick latency (TFL) assay, as detailed previously (Lewis et al., 1991; Henderson et al., 2014; Gaston et al., 2021; Jenkins et al., 2021). Prior to administration of fentanyl, the rats were allowed to crawl inside a canvas garden glove and lightly restrained within the glove to allow for the thermal withdrawal latencies to be determined. After injection of fentanyl, placement of the rat in the glove was aided by an investigator. Each investigator performing the TFL assay was unaware of the treatments that the rats were then subjected to. The TFL testing apparatus consisted of a beam of focused radiant heat provided by a 50 W projector lamp, which was focused on the underside of the tail at 1 of 5 sites 8–10 mm apart. TFL was measured to the nearest 0.1 s as the time from onset of heating of the tail to withdrawal of the tail from the heat. The intensity of the light beam was set so that baseline TFL values were about 2.5 s. A cutoff time of 12 s was set to minimize potential damage to the tail upon repeated application of the radiant heat beam. TFL was established before and during the various stages of the experiment. The data are presented as actual TFL values (sec), arithmetic changes (sec), and as maximum possible effect (%MPE) using the formula, %MPE = [(post-injection TFL − baseline TFL)/(12 − baseline TFL)] × 100. A description of the protocols undergone by the five treatment groups of rats used and their body weights are provided in Supplementary Table S1. Group 1 rats received a continuous infusion of phosphate-buffered saline (PBS) (i.e., vehicle) at pH 7.2. After 60 min these rats received three injections of vehicle, each given 30 min apart. Thirty minutes after injection of the third injection of vehicle, the rats received an injection of NLXmi (2.5 mg/kg, IV). Group 2 rats received a continuous infusion of vehicle and after 60 min, injections of fentanyl (10, 25, and 50 μg/kg), each given 30 min apart. Thirty minutes after injection of the 50 μg/kg dose of fentanyl, the rats received an injection of vehicle (100 μl/kg, IV). Group 3–5 rats received a continuous infusion of L-CSNO (200 nmol/kg/min, IV) or L-cysteine (200 nmol/kg/min, IV) or D-CSNO (200 nmol/kg/min, IV), respectively and after 60 min, injections of fentanyl (10, 25, and 50 μg/kg) each given 30 min apart. Thirty minutes after injection of the 50 μg/kg dose of fentanyl, the rats then received an injection of NLXmi (2.5 mg/kg, IV). It is important to note that the infusions of the test agents were continued throughout the entire protocol (i.e., until 15 min after the injection of NLXmi or vehicle).

### Data Analyses

All data are presented as mean ± SEM and were evaluated using one-way and two-way ANOVA followed by Bonferroni corrections for multiple comparisons between means using the error mean square terms from each ANOVA analysis (Winer, 1971; Wallenstein et al., 1980; Ludbrook, 1998; McHugh, 2011) as detailed previously (Getsy et al., 2021; Jenkins et al., 2021). A value of *p* < 0.05 was taken as the initial level of statistical significance (Wallenstein et al., 1980; Ludbrook, 1998; McHugh, 2011). Statistical analyses were performed using GraphPad Prism software (GraphPad Software, Inc., La Jolla, CA, United States). A detailed description of these statistical procedures is provided in the Supplementary Material under “Detailed description of Statistical Approaches.”

## Results

### SNO-L-CYS Infusion Blunts the Fentanyl-Induced Changes in f_R_, V_T_ and V_E_

**FIGURE 1:**
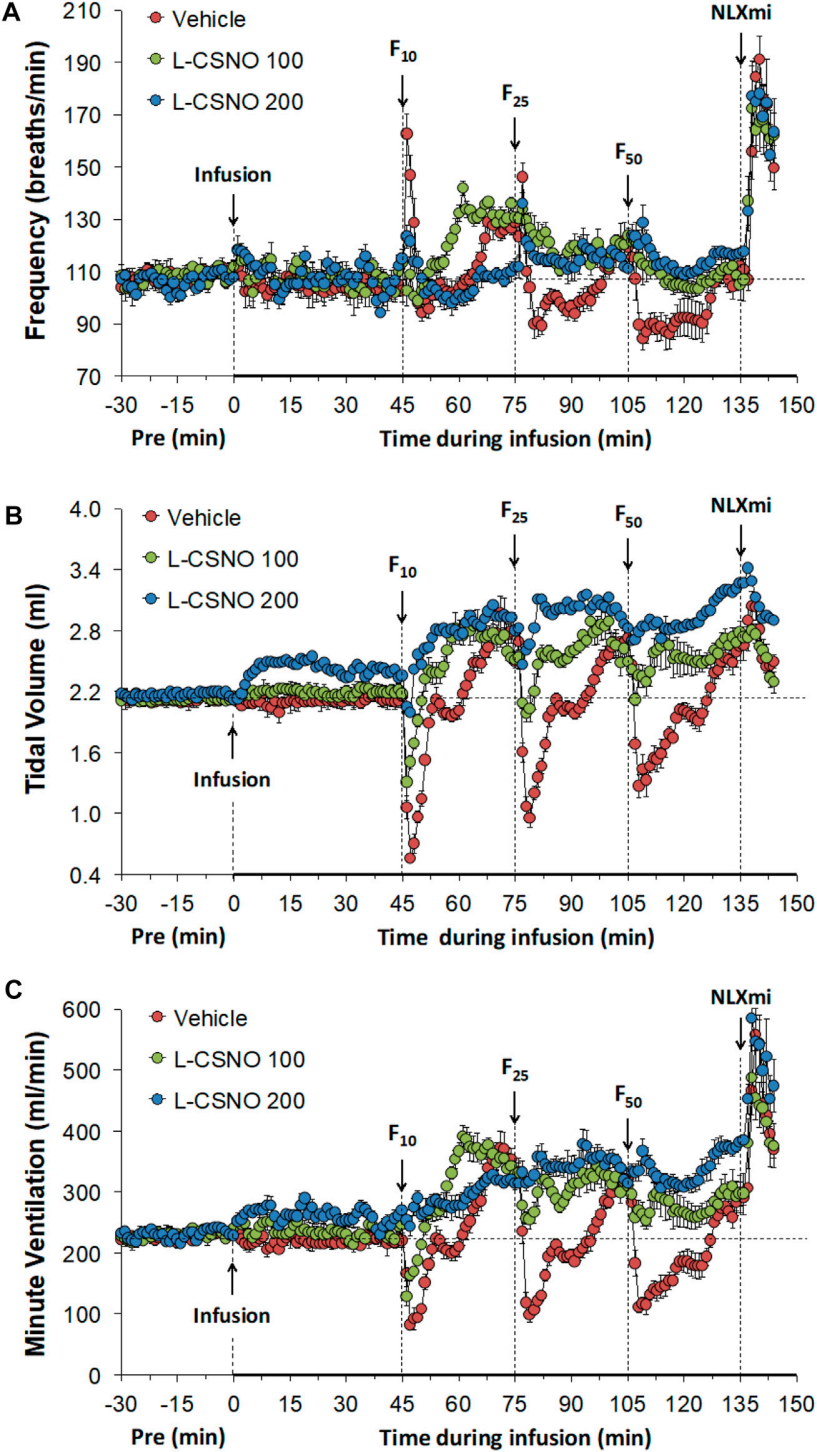
Frequency of breathing **(A)**, tidal volume **(B)** and minute ventilation **(C)** values. The intravenous infusion of vehicle (20 μl/min, IV), L-CSNO 100 (S-nitroso-L-cysteine, 100 nmol/kg/min, IV) or L-CSNO 200 (S-nitroso-L-cysteine, 200 nmol/kg/min, IV) began at time 0. Bolus injections of fentanyl at F10 (10 μg/kg, IV), F25 (25 μg/kg, IV), and F50 (50 μg/kg, IV) were given at 45, 75, and 105 min, respectively. A bolus intravenous injection of naloxone methiodide (NLXmi, 1.5 mg/kg, IV) was given at time 135 min. Data are presented as mean ± SEM. There were 8 rats in each group.

The actual f_R_, V_T_, and V_E_ values during various stages of the L-CSNO (100 or 200 nmol/kg/min, IV) infusion experiments are presented in Figure 1. As seen in Figure 1A, the infusions of vehicle or L-CSNO at 100 (L-CSNO 100) or 200 (L-CSNO 200) nmol/kg/min did not alter resting levels of f_R_. In vehicle-infused rats, injection of the 10 μg/kg dose of fentanyl elicited a transient rise in f_R_ that quickly subsided before rising again to a plateau level that was sustained for the final 5 min of the post-injection period. Subsequent injection of the 25 μg/kg dose of fentanyl caused a transient rise in f_R_ in the vehicle-infused rats that rapidly fell to below baseline levels, and then gradually recovered to values above pre-injection between 25 and 30 min post-injection. The ensuing injection of the 50 μg/kg dose of fentanyl caused an immediate fall in f_R_ in the vehicle-infused rats that was sustained for about 20–25 min before returning to baseline values. As is apparent from of Figure 1A, the ability of the injections of fentanyl to decrease f_R_ was substantially diminished in rats receiving the 100 or 200 nmol/kg/min infusions of L-CSNO. The dramatic effects elicited by the subsequent injection of NLXmi will be detailed below (see section *Profound effects of NLXmi in fentanyl-injected rats*). As seen in Figure 1B, the infusions of vehicle or L-CSNO 100 did not alter V_T_, whereas infusion of L-CSNO 200 elicited a relatively minor, but sustained increase in V_T_. The injection of 10 μg/kg of fentanyl elicited a marked decrease in V_T_ for about 5 min in the vehicle-infused and L-CSNO 100 rats, and that was followed by a sustained increase in V_T_. The injection of 10 μg/kg of fentanyl caused V_T_ of the L-CSNO 200 rats to decrease slightly for about 5 min, and that also was followed by a sustained increase. Subsequent injections of 25 and 50 μg/kg fentanyl elicited qualitatively similar responses to the 10 μg/kg dose of fentanyl in vehicle-infused rats, although the duration of the decrease in V_T_ was somewhat larger after injection of the 50 μg/kg dose of fentanyl in vehicle-infused rats. The 25 and 50 μg/kg injections of fentanyl elicited markedly smaller decreases in V_T_ in rats receiving the 100 or 200 nmol/kg/min infusions of L-CSNO, and V_T_ remained above baseline levels for most of the recording period in these two groups. As seen in of Figure 1C, infusions of vehicle or 100 nmol/kg/min of L-CSNO did not alter V_E_, whereas infusion of L-CSNO at 200 nmol/kg/min elicited a minor, but sustained increase in V_E_. The injection of the 10 μg/kg dose of fentanyl elicited a pronounced decrease in V_E_ of about 5 min in duration in the vehicle-infused and L-CSNO 100 rats that was followed by a substantial and sustained rise in V_E_ above baseline values. V_E_ of L-CSNO 200 rats did not change after injection of 10 μg/kg fentanyl, but did gradually increase approximately 20 min post-injection of 10 μg/kg dose of fentanyl even higher above baseline. Injections of 25 and 50 μg/kg of fentanyl elicited qualitatively similar responses in vehicle-infused rats, although the decrease in V_E_ lasted somewhat longer after injection of 50 μg/kg fentanyl in vehicle-infused rats. The injections of 25 and 50 μg/kg doses of fentanyl elicited smaller decreases in V_E_ in rats that were receiving the 100 nmol/kg/min infusion of L-CSNO compared to vehicle-infused rats, and we saw no decreases in V_E_ in rats that were receiving the 200 nmol/kg/min infusion of L-CSNO. Thus, except for the decrease in V_E_ elicited by 10 μg/kg fentanyl in rats receiving 100 nmol/kg/min of L-CSNO, V_E_ remained above baseline levels for the post-injection periods for the L-CSNO 100 and L-CSNO 200 groups.

**FIGURE 2:**
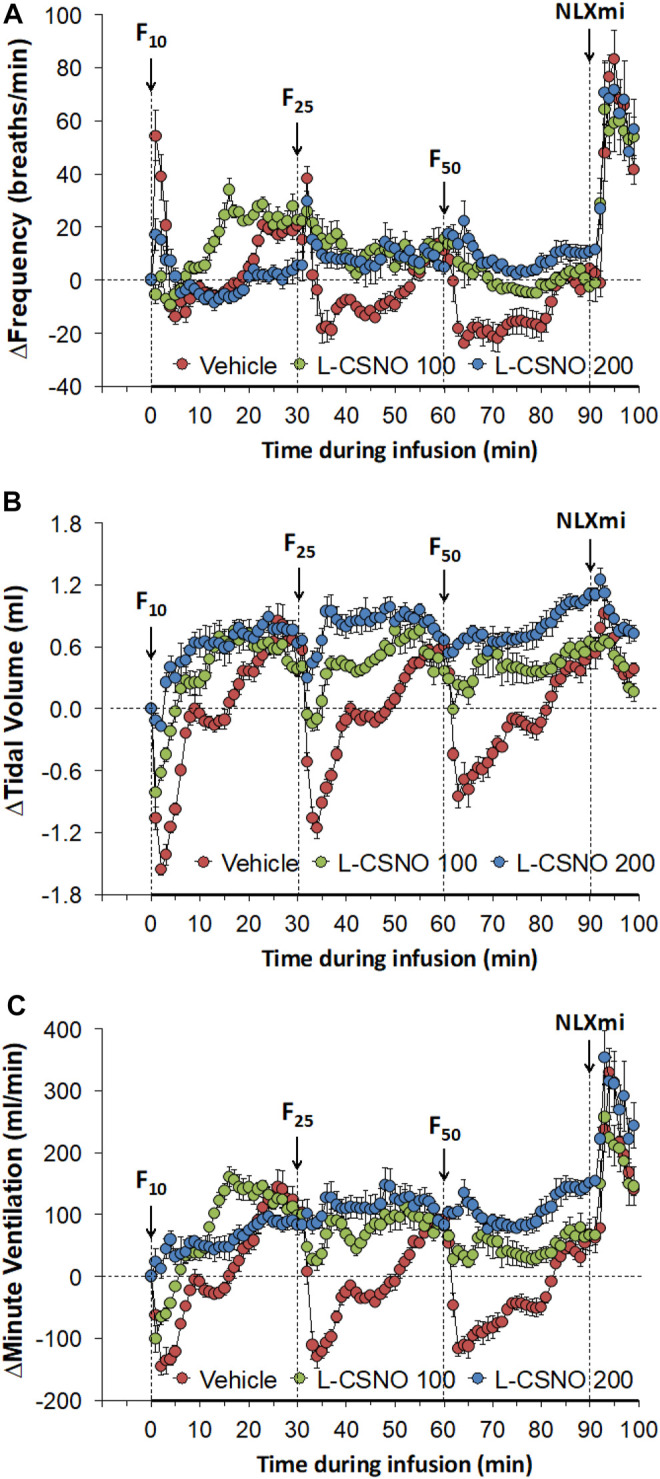
Arithmetic changes in baseline frequency of breathing **(A)**, tidal volume **(B)** and minute ventilation **(C)** elicited by bolus injections of fentanyl at F10 (10 μg/kg, IV), F25 (25 μg/kg, IV), and F50 (50 μg/kg, IV) in rats receiving continuous infusion of vehicle (20 μl/min, IV), L-CSNO 100 (S-nitroso-L-cysteine, 100 nmol/kg/min, IV) or L-CSNO 200 (S-nitroso-L-cysteine, 200 nmol/kg/min, IV). A bolus injection of naloxone methiodide (NLXmi (2.5 mg/kg, IV) was given at the 90 min timepoint. The data are presented as mean ± SEM. There were 8 rats in each group.

**FIGURE 3:**
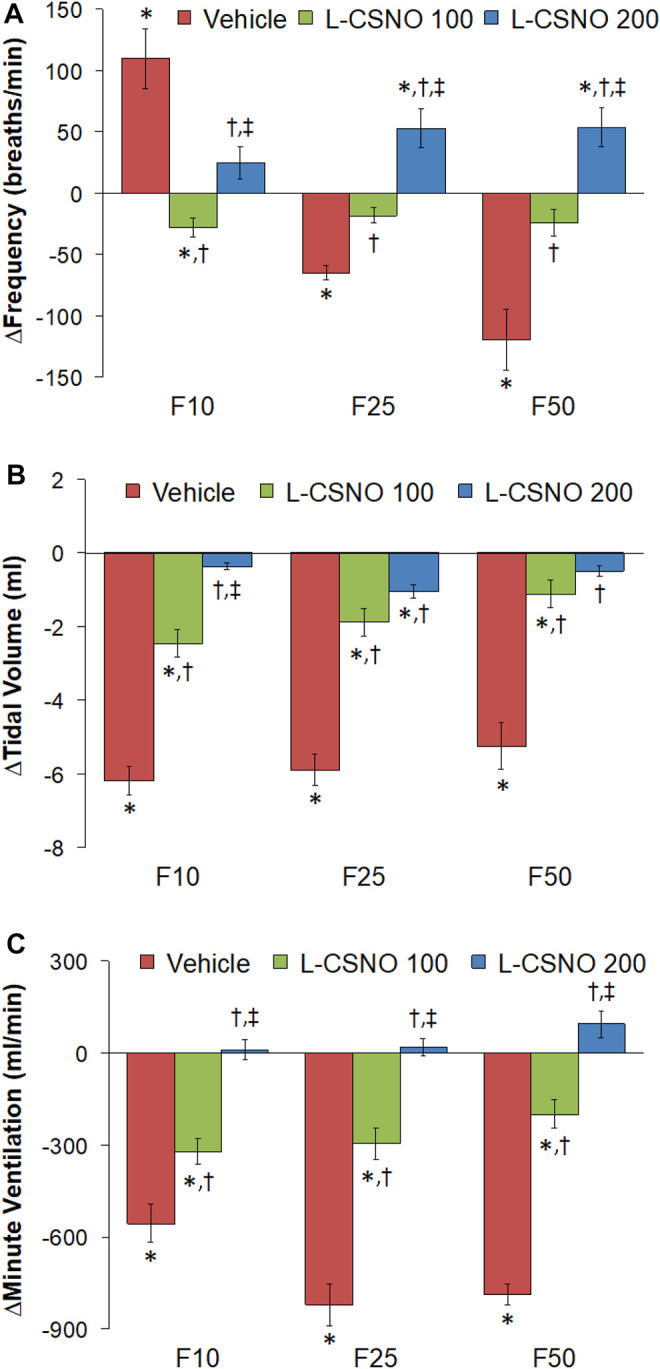
Total arithmetic changes in baseline frequency of breathing **(A)**, tidal volume **(B)**, and minute ventilation **(C)** during the first 5 min following injection of fentanyl at F10 (10 μg/kg, IV), F25 (25 μg/kg, IV), and F50 (50 μg/kg, IV) in rats receiving infusion of vehicle (20 μl/min, IV), L-CSNO 100 (S-nitroso-L-cysteine, 100 nmol/kg/min, IV) or L-CSNO 200 (S-nitroso-L-cysteine, 200 nmol/kg/min, IV). The data are presented as mean ± SEM. There were 8 rats in each group. *p < 0.05, significant change from Pre values. ^†^ *p* < 0.05, L-CSNO 100 or L-CSNO 200 versus vehicle. ^‡^ *p* < 0.05, L-CSNO 200 versus L-CSNO 100.

The arithmetic changes in f_R_, V_T_, and V_E_ (expressed as differences from the 45 min infusion timepoint) shown in Figure 2, confirm that the ability fentanyl to adversely affect f_R_, V_T_, and V_E_ was markedly diminished in rats receiving infusions of L-CSNO 100 and L-CSNO 200. Figure 3 summarizes the total (cumulative) arithmetic changes in f_R_ (Figure 3A), V_T_ (Figure 3B) and V_E_ (Figure 3C) from baseline (Pre) values during the first 5 min following injection of fentanyl at 10 μg/kg (F10), 25 μg/kg (F25), and 50 μg/kg (F50) in rats receiving infusion of vehicle (20 μl/min, IV), L-CSNO at 100 nmol/kg/min (L-CSNO 100) or 200 nmol/kg/min (L-CSNO 200). In vehicle-infused rats, injection of the 10 μg/kg dose of fentanyl caused a cumulative increase in f_R_, whereas the 25 and 50 μg/kg doses of fentanyl caused cumulative decreases in f_R_. The increase in f_R_ elicited by the 10 μg/kg dose of fentanyl, and the decreases in f_R_ elicited by the 25 and 50 μg/kg doses in vehicle-infused rats were diminished in rats receiving the 100 nmol/kg/min infusion of L-CSNO. The increase in f_R_ elicited by the 10 μg/kg dose of fentanyl in vehicle-infused rats was smaller in rats receiving 200 nmol/kg/min infusion of L-CSNO, and the decreases in f_R_ elicited by the 25 and 50 μg/kg doses of fentanyl were reversed to increases in f_R_ in the L-CSNO 200 rats. All doses of fentanyl elicited substantial total falls in V_T_ and V_E_ in rats receiving the infusion of vehicle. These decreases in V_T_ and V_E_ were markedly smaller in the rats receiving the infusion of L-CSNO 100. In the rats receiving L-CSNO 200, the decreases in V_T_ were even smaller than L-CSNO 100, and the V_E_ was slightly increased over the 3 doses of fentanyl in this group due to the increases in f_R_.

**FIGURE 4:**
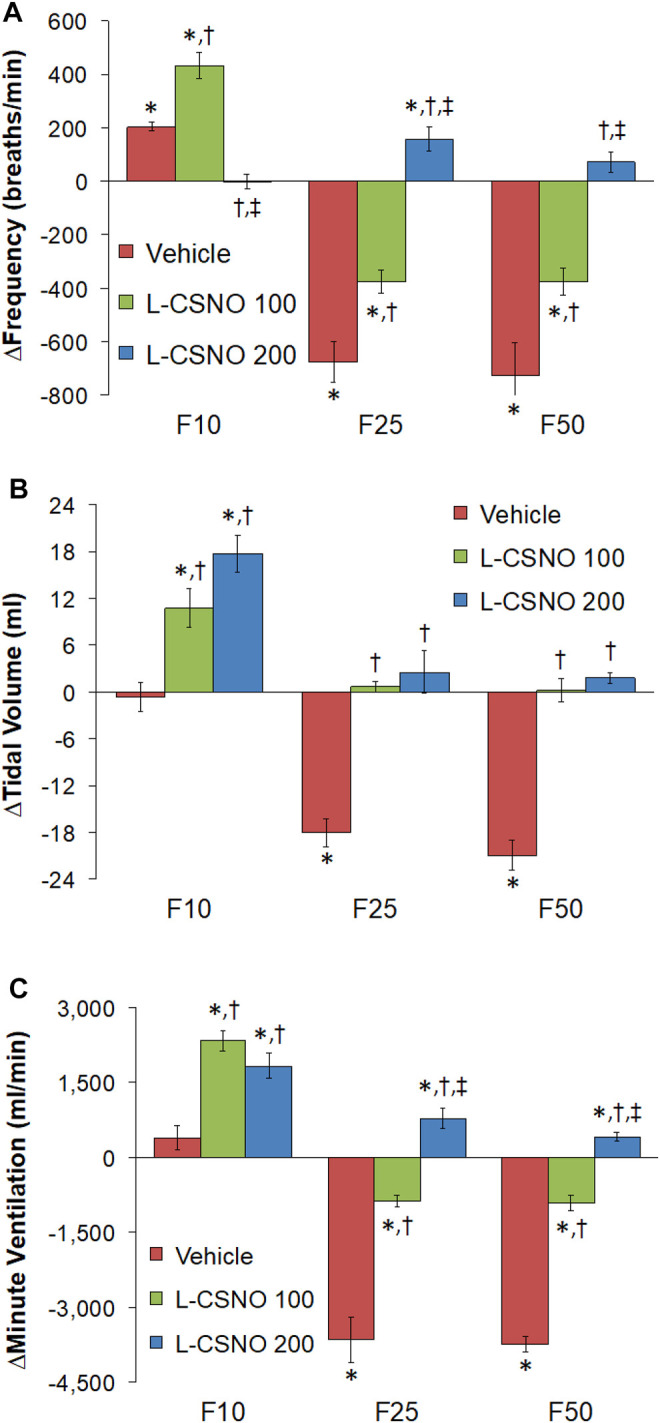
Total arithmetic changes in baseline frequency of breathing **(A)**, tidal volume **(B)**, and minute ventilation **(C)** during the 30 min period following injection of fentanyl at F10 (10 μg/kg, IV), F25 (25 μg/kg, IV) and F50 (50 μg/kg, IV) in rats receiving intravenous infusion of vehicle (20 μl/min, IV), L-CSNO 100 (S-nitroso-L-cysteine, 100 nmol/kg/min, IV) or L-CSNO 200 (S-nitroso-L-cysteine, 200 nmol/kg/min, IV). The data are presented as mean ± SEM. There were 8 rats in each group. *p < 0.05, significant change from Pre values. ^†^ *p* < 0.05, L-CSNO 100 or L-CSNO 200 versus vehicle. ^‡^ *p* < 0.05, L-CSNO 200 versus L-CSNO 100.

Figure 4 summarizes the total arithmetic changes in f_R_ (Figure 4A), V_T_ (Figure 4B) and V_E_ (Figure 4C) from baseline (Pre) values during the entire 30 min period after injection of fentanyl at 10 μg/kg (F10), 25 μg/kg (F25), and 50 μg/kg (F50) in rats receiving infusion of vehicle (20 μl/min, IV), L-CSNO at 100 nmol/kg/min (L-CSNO 100) or 200 nmol/kg/min (L-CSNO 200). The 10 μg/kg dose of fentanyl elicited a cumulative increase in f_R_, whereas the 25 and 50 μg/kg doses of fentanyl caused cumulative decreases in f_R_ in the vehicle-infused rats. The increase in f_R_ elicited by 10 μg/kg fentanyl in vehicle-infused rats was augmented, and the decreases in f_R_ elicited by the 25 and 50 μg/kg doses of fentanyl were diminished in rats receiving the 100 nmol/kg/min infusion of L-CSNO. The changes in f_R_ elicited by 10, 25, and 50 μg/kg fentanyl were markedly diminished or slightly increased in the rats receiving the 200 nmol/kg/min infusion of L-CSNO, respectively. The changes in V_T_ and V_E_ elicited by the 10 μg/kg dose of fentanyl in vehicle-infused rats were minor compared to the pronounced increases in V_T_ and V_E_ in rats receiving the 100 or 200 nmol/kg/min infusions of L-CSNO. The decreases in V_T_ elicited by the 25 and 50 μg/kg doses of fentanyl in vehicle-infused rats were either increased or abolished in rats receiving the 100 or 200 nmol/kg/min infusions of L-CSNO. Additionally, the decreases in V_E_ elicited by the 25 and 50 μg/kg doses of fentanyl in vehicle-infused rats were markedly diminished in rats receiving the 100 nmol/kg/min infusion of L-CSNO, and increased in rats receiving the 200 nmol/kg/min infusion of L-CSNO.

Table 2 summarizes the values of ventilatory parameters at various stages of the L-CSNO infusion experiments. The baseline values (i.e., those prior to commencing the infusions) for f_R_, V_T_, and V_E_ were similar to one another (*p* > 0.05, for all comparisons). The infusion of L-CSNO at 100 nmol/kg/min did not affect f_R_, V_T_, and V_E_ compared to vehicle (Figure 1), thus the f_R_, V_T_, and V_E_ values recorded at 45 min post start of infusion (i.e., pre-F10 values—or the values prior to the injection of the 10 μg/kg dose of fentanyl) were similar in the L-CSNO 100 group of rats compared to vehicle (Table 2). In contrast, the infusion of L-CSNO at 200 nmol/kg/min elevated V_T_ and V_E_, whereas it did not change f_R_, for the pre-F10 values (Table 2; Figure 1). Additionally, both Table 2 and Figure 1 show that the values of f_R_, V_T_, and V_E_ were elevated equally immediately prior to injection of the 25 and 50 μg/kg doses of fentanyl in the rats receiving the infusions of vehicle or L-CSNO (100 or 200 nmol/kg/min, IV), with the exception of f_R_ for the L-CSNO 200 group immediately prior to injection of the 25 μg/kg dose of fentanyl, in which f_R_ was similar to baseline. Therefore, these elevations in ventilatory parameters were evidently due to the injections of fentanyl with minimal differences occurring because of the presence of L-CSNO.

**TABLE 2:** Baseline parameters at the beginning of the experiments (Pre) and prior to each injection of fentanyl (F10, F25, and F50) in the L-CSNO infusion experiments.

Parameter	Group	Baseline	Pre-F10	Pre-F25	Pre-F50
Frequency, breaths/min	Vehicle	108 ± 3	104 ± 4	128 ± 3*	121 ± 7*
	L-CSNO 100	108 ± 2	108 ± 3	132 ± 2*	121 ± 2*
	L-CSNO 200	106 ± 2	111 ± 4	110 ± 4	113 ± 2
Tidal Volume, ml	Vehicle	2.12 ± 0.03	2.12 ± 0.07	2.68 ± 0.08*	2.69 ± 0.10*
	L-CSNO 100	2.13 ± 0.03	2.20 ± 0.05	2.54 ± 0.07*	2.53 ± 0.13*
	L-CSNO 200	2.17 ± 0.02	2.37 ± 0.04^*^	2.29 ± 0.08*	2.88 ± 0.07*
Minute Ventilation, ml/min	Vehicle	229 ± 6	220 ± 10	342 ± 14*	320 ± 9*
	L-CSNO 100	230 ± 6	237 ± 3	337 ± 9*	309 ± 19*
	L-CSNO 200	231 ± 6	263 ± 10*	317 ± 9*	325 ± 10*
NEBI, % of epoch	Vehicle	3.3 ± 0.4	2.8 ± 0.4	2.9 ± 1.0	3.3 ± 0.6
	L-CSNO 100	3.1 ± 0.2	3.7 ± 0.6	2.9 ± 0.5	3.0 ± 0.4
	L-CSNO 200	3.5 ± 0.2	3.2 ± 0.4	3.0 ± 0.2	2.4 ± 0.1*
[(NEBI, %)/Frequency, bpm)] × 100	Vehicle	3.0 ± 0.3	2.6 ± 0.4	2.2 ± 0.8	2.7 ± 0.5
	L-CSNO 100	2.9 ± 0.1	3.5 ± 0.6	2.3 ± 0.4	2.6 ± 0.3
	L-CSNO 200	3.4 ± 0.2	2.8 ± 0.3	2.8 ± 0.3	2.2 ± 0.1*

Frequency, frequency of breathing; NEBI, non-eupneic breathing index; bpm, breaths per minute; L-CSNO, S-nitroso-L-cysteine; F10, F25, and F50, fentanyl at intravenous doses of 10, 25, and 50 μg/kg, respectively. The data are presented as mean ± SEM. There were 8 rats in each group. *p < 0.05, significant change from Pre values.

**FIGURE 5:**
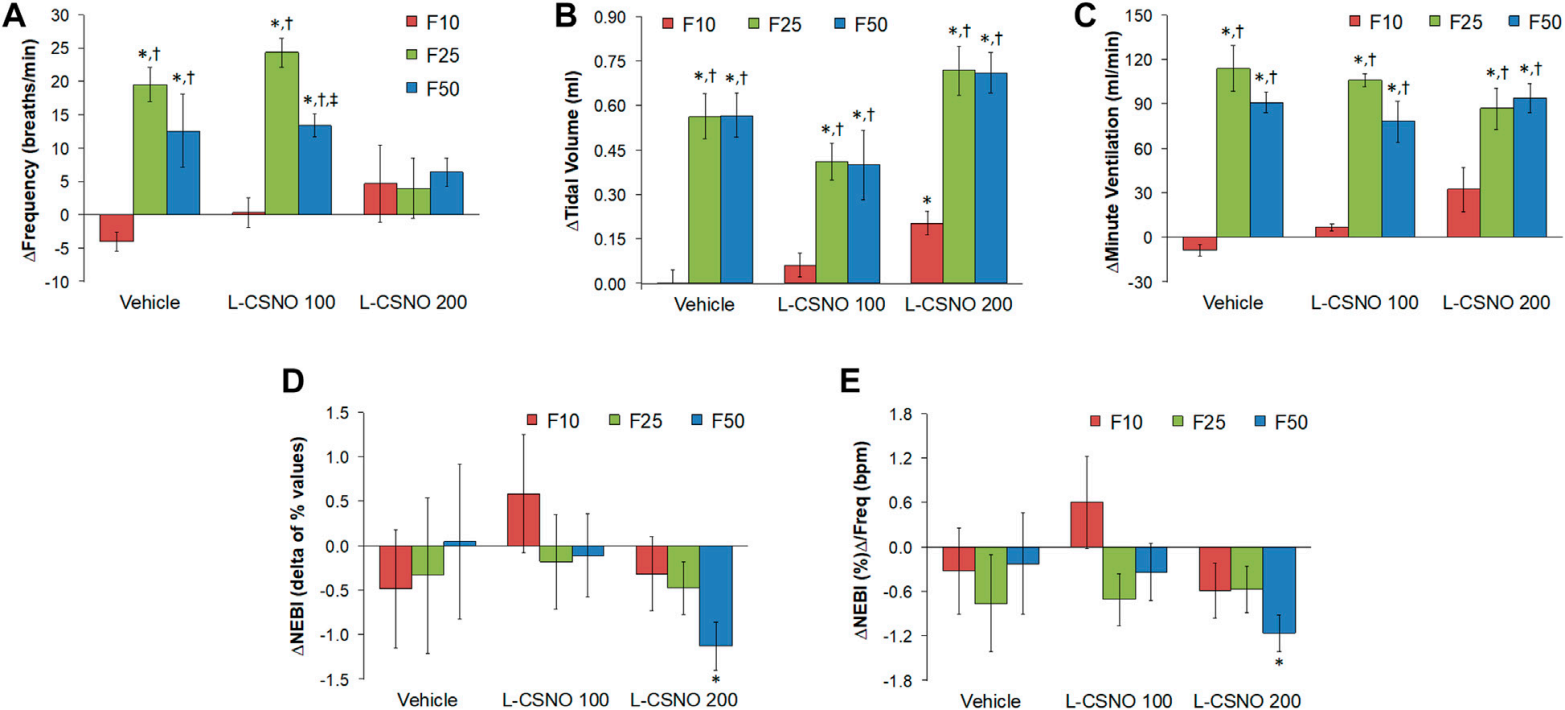
Arithmetic changes in Pre values (i.e., those prior to any drug administration) from values just before injection of fentanyl at F10 (10 μg/kg, IV), F25 (25 μg/kg, IV), and F50 (50 μg/kg, IV) in rats that were receiving infusion of vehicle (20 μl/min, IV), L-CSNO 100 (S-nitroso-L-cysteine, 100 nmol/kg/min, IV) or L-CSNO 200 (S-nitroso-L-cysteine, 200 nmol/kg/min, IV). **(A)** Frequency of breathing. **(B)** Tidal volume. **(C)** Minute ventilation. **(D)** Non-eupneic breathing index (NEBI). **(E)** NEBI/Frequency of breathing (NEBI/Freq). The data are presented as mean ± SEM. There were 8 rats in each group. *p < 0.05, significant change from Pre values. ^†^ *p* < 0.05, F25 or F50 versus F10. ^‡^ *p* < 0.05, L-CSNO 200 versus L-CSNO 100.

The arithmetic changes in Pre values (i.e., those prior to any drug administration) from values just before injection of fentanyl at F10 (10 μg/kg, IV), F25 (25 μg/kg, IV), and F50 (50 μg/kg, IV) in rats receiving infusion of vehicle (20 μl/min, IV), L-CSNO 100 (S-nitroso-L-cysteine, 100 nmol/kg/min, IV) or L-CSNO 200 (S-nitroso-L-cysteine, 200 nmol/kg/min, IV) are provided in Figure 5. We see significant arithmetic changes for f_R_, V_T_, and V_E_ with the vehicle and L-CSNO 100 infusions for F25 and F50. We see no significant changes for f_R_ with the L-CSNO 200 infusion for F25 and F50, and no significant changes for V_E_ with the L-CSNO 200 infusion for F10. Nevertheless, we do see significant arithmetic changes for V_T_ with the L-CSNO 200 infusion for F10, F25, and F50, and significant arithmetic changes for V_E_ with the L-CSNO 200 infusion for F25 and F50. Additionally, we see no significant changes for f_R_, V_T_ and V_E_ values for F10 across all three continuous infusions, except V_T_ values with the L-CSNO 200 infusion. The levels of f_R_ prior to injections of the 25 and 50 μg/kg doses of fentanyl were higher than baseline infusion values in rats receiving the 100, but not the 200 nmol/kg/min continuous infusions of L-CSNO. In contrast, the levels of V_T_ and V_E_ prior to the injections of the 25 and 50 μg/kg doses of fentanyl were higher than baseline infusion values in the rats receiving both the 100 or 200 nmol/kg/min infusions of L-CSNO. The cumulative changes in ventilatory parameters elicited by continuous intravenous infusion of vehicle or L-CSNO (L-CSNO 100 and L-CSNO 200) during the 45 min of infusion are shown in Table 3. The values confirm that the infusion of L-CSNO at 200 nmol/kg/min, but not the 100 nmol/kg/min, caused significant cumulative increases in V_T_ and V_E_, but not f_R_.

**TABLE 3:** Cumulative changes in Pre ventilatory parameters elicited by the continuous intravenous infusion of vehicle or S-nitroso-L-cysteine (L-CSNO) during the 45 min of infusion.

Parameter	Vehicle	L-CSNO 100	L-CSNO 200
Frequency, %	−3.1 ± 2.3	−0.3 ± 1.2	+2.2 ± 3.7
Tidal Volume, %	−0.6 ± 1.6	+2.7 ± 2.1	+11.6 ± 0.8*^,†^
Minute Ventilation, %	−3.9 ± 1.3*	+2.2 ± 1.0	+14.1 ± 4.7*^,†^
NEBI, %	−11.7 ± 2.2*	−41.5 ± 4.7*^,†^	−33.5 ± 3.1*^,†^
(NEBI)/Frequency, %	−9.2 ± 1.3*	−41.4 ± 4.9*^,†^	−36.0 ± 3.9*^,†^

Frequency, frequency of breathing; NEBI, non-eupneic breathing index; L-CSNO 100 or L-CSNO 200, S-nitroso-L-cysteine given intravenously at 100 or 200 nmol/kg/min. The data are presented as mean ± SEM. There were 8 rats in each group. *p < 0.05, significant change from Pre values. ^†^ *p* < 0.05, L-CSNO 100 or L-CSNO 200 versus vehicle.

### Infusions of L-Cysteine or D-CSNO Do Not Blunt the Fentanyl-Induced Changes in f_R_, V_T_, and V_E_

**FIGURE 6:**
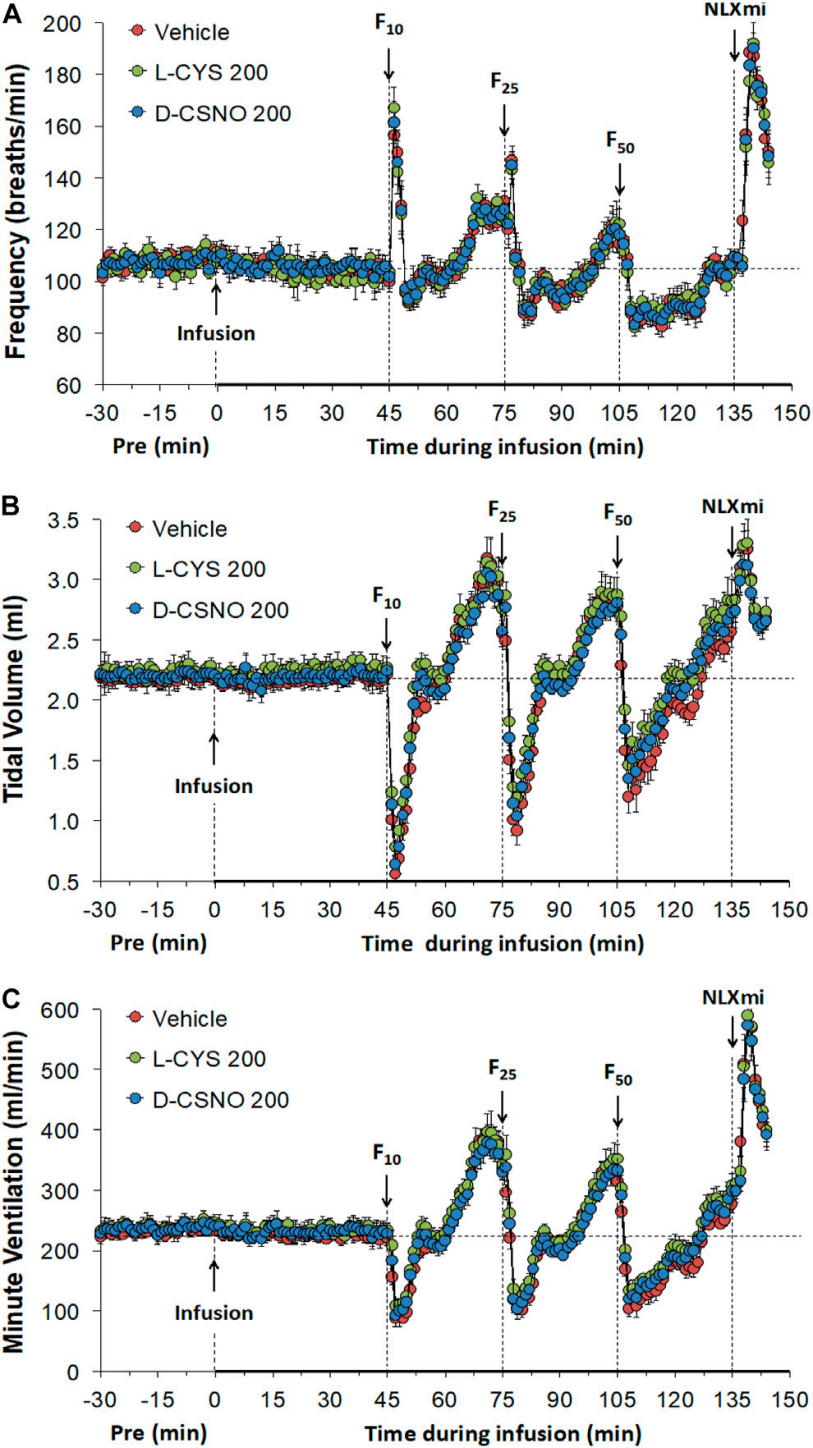
Frequency of breathing **(A)**, tidal volume **(B)** and minute ventilation **(C)** values. The infusion of vehicle (20 μl/min, IV), L-cysteine (200 nmol/kg/min, IV) or S-nitroso-D-cysteine (D-CSNO, 200 nmol/kg/min, IV) began at time 0. Bolus injections of fentanyl at F10 (10 μg/kg, IV), F25 (25 μg/kg, IV), and F50 (50 μg/kg, IV) were given at 45, 75, and 105 min, respectively. A bolus intravenous injection of naloxone methiodide (NLXmi, 1.5 mg/kg, IV) was given at time 135 min. Data are presented as mean ± SEM. There were 8 rats in each group.

**FIGURE 7:**
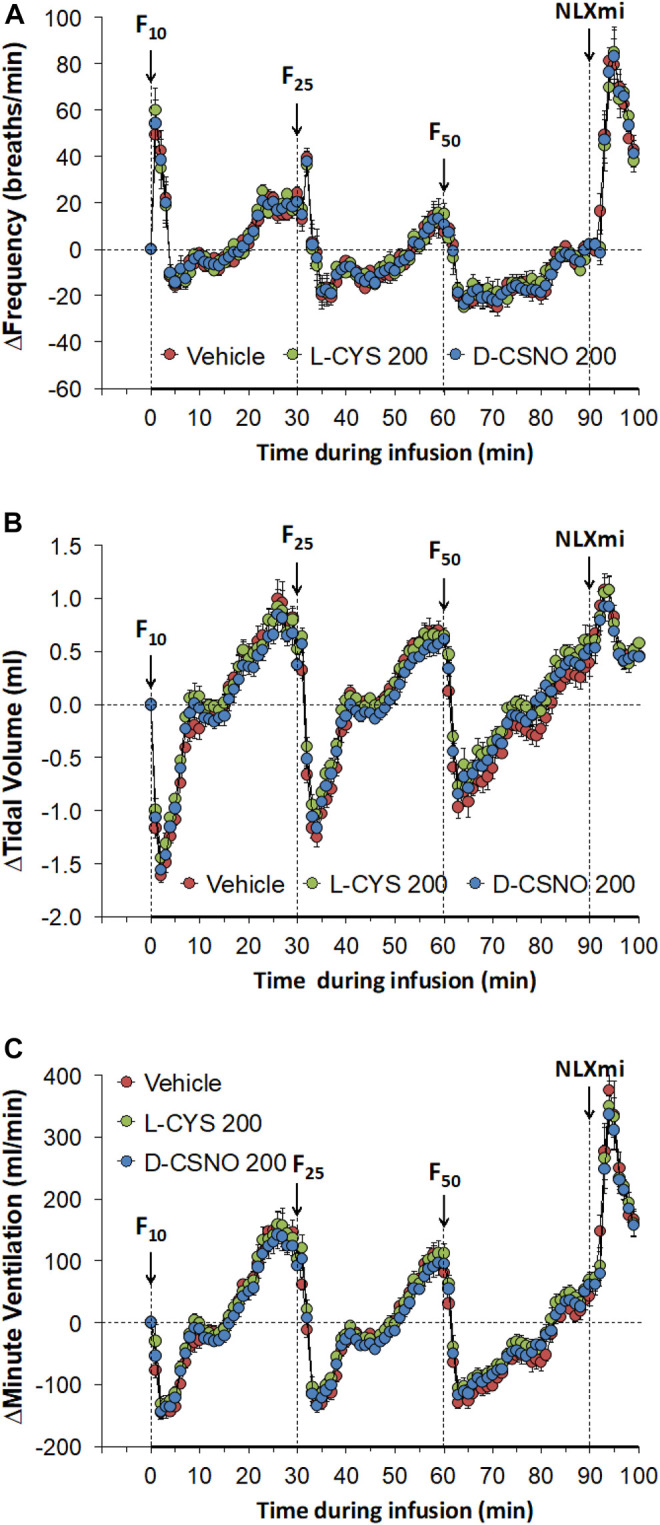
Arithmetic changes in baseline frequency of breathing **(A)**, tidal volume **(B)** and minute ventilation **(C)** elicited by injections of fentanyl at F10 (10 μg/kg, IV), F25 (25 μg/kg, IV), and F50 (50 μg/kg, IV) in rats receiving continuous infusion of vehicle (20 μl/min, IV), L-cysteine (200 nmol/kg/min, IV) or S-nitroso-D-cysteine (D-CSNO, 200 nmol/kg/min, IV). The data are presented as mean ± SEM. There were 8 rats in each group.

**FIGURE 8:**
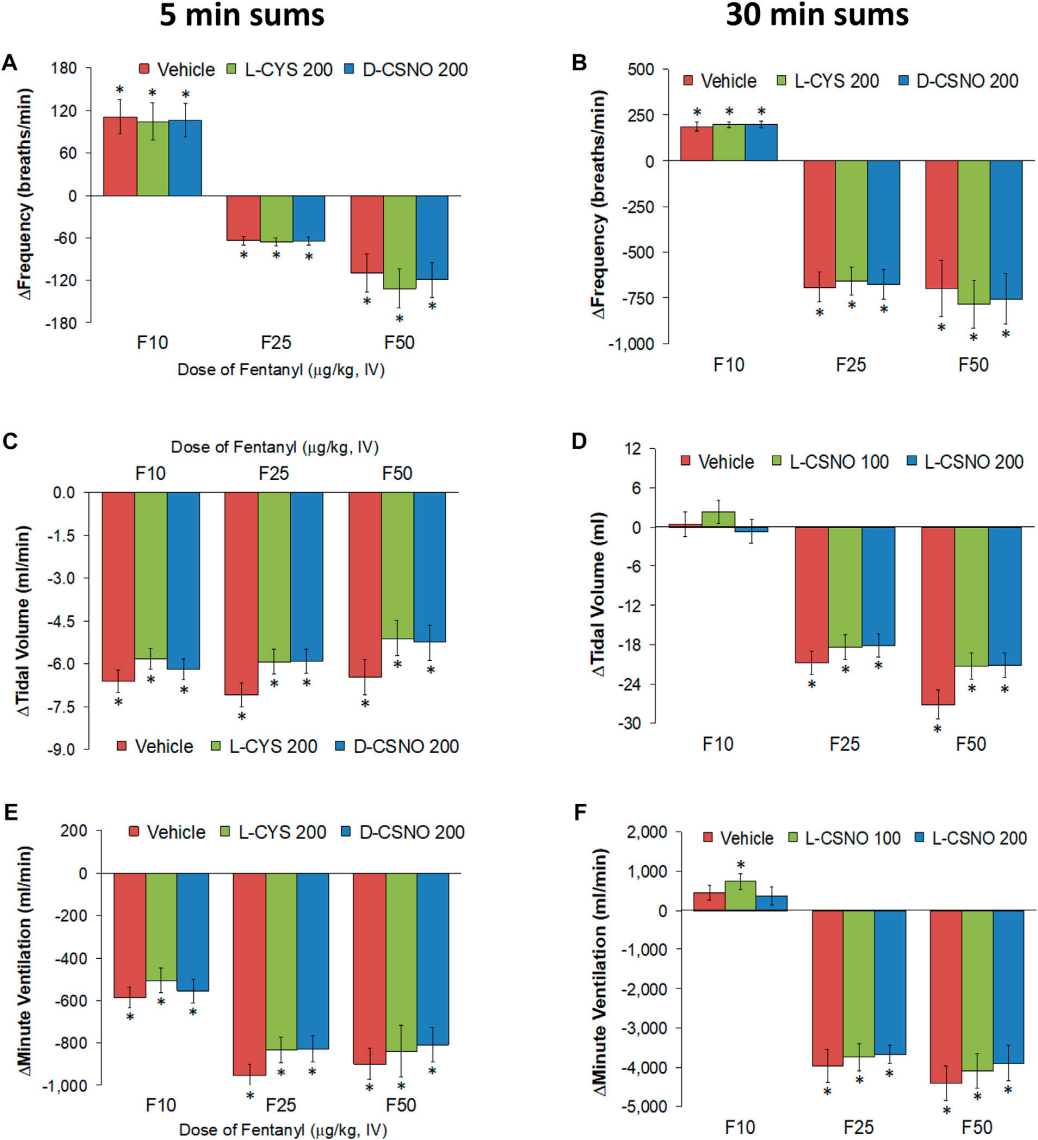
Total (cumulative) arithmetic changes in Pre values during the first 5 min and entire 30 min periods in frequency of breathing **(A,B)**, tidal volume **(C,D)**, and minute ventilation **(E,F)** after injection of fentanyl at F10 (10 μg/kg, IV), F25 (25 μg/kg, IV), and F50 (50 μg/kg, IV) in rats receiving infusion of vehicle (20 μl/min, IV), L-cysteine (200 nmol/kg/min) or D-CSNO (200 nmol/kg/min). The data are presented as mean ± SEM. There were 8 rats in each group. *p < 0.05, significant change from Pre values.

Figure 6 shows the values for f_R_ (Figure 6A), V_T_ (Figure 6B), and V_E_ (Figure 6C) during studies involving injections of fentanyl (10, 25, and 50 μg/kg, IV) in rats receiving infusions of vehicle (20 μl/min, IV), L-cysteine (200 nmol/kg/min, IV) or D-CSNO (200 nmol/kg/min, IV). The infusions of L-cysteine or D-CSNO did not alter baseline values. The injections of fentanyl at 10 μg/kg (F10), 25 μg/kg (F25), and 50 μg/kg (F50) elicited very similar qualitative and quantitative responses to those of vehicle-infused rats described in Figure 1. Thus, the fentanyl responses at 10, 25, and 50 μg/kg are not affected by continuous infusion of L-cysteine or D-CSNO. The arithmetic changes in f_R_, V_T_, and V_E_ (expressed as changes from the 45 min infusion timepoint) shown in Figure 7, also confirm that the continuous infusions of L-cysteine or D-CSNO do not change the effects of fentanyl on f_R_, V_T_, and V_E_ compared to vehicle-infused rats. Figure 8 summarizes the total (cumulative) arithmetic changes from Pre values during the first 5 min and entire 30 min recording periods in f_R_ (Figures 8A,B, respectively), V_T_ (Figures 8C,D, respectively), and V_E_ (Figures 8E,F, respectively) after injection of fentanyl at F10 (10 μg/kg, IV), F25 (25 μg/kg, IV), and F50 (50 μg/kg, IV) in rats receiving infusion of vehicle (20 μl/min, IV), or L-cysteine or D-CSNO at 200 nmol/kg/min. The total increases in f_R_ over the first 5 min elicited by the 10 μg/kg dose of fentanyl was similar in the three groups as were the total decreases in f_R_ elicited by the 25 and 50 μg/kg doses of fentanyl. The total decreases in V_T_ and V_E_ over the first 5 min elicited by the 10, 25, and 50 μg/kg doses of fentanyl were similar in all three groups. Additionally, similar cumulative changes in f_R_, V_T_, and V_E_ were observed over the entire 30 min recording period in the three groups at the 25 and 50 μg/kg doses of fentanyl. At the 10 μg/kg dose of fentanyl, changes in f_R_, V_T_, and V_E_ were increased or not changed from Pre values for the three groups. Supplementary Table S2 summarizes the values of ventilatory parameters during the L-cysteine and D-CSNO infusion studies. Baseline values (Pre—those before commencing the infusions) for f_R_, V_T_, and V_E_ were similar to one another (*p* > 0.05, for all comparisons). The infusion of L-cysteine or D-CSNO at 200 nmol/kg/min did not affect f_R_, V_T_, and V_E_ at the 45 min infusion timepoint (Pre-F10 values—those prior to injection of the 10 μg/kg dose of fentanyl). The f_R_, V_T_, and V_E_ values prior to the injection of the 25 and 50 μg/kg doses of fentanyl (Pre-F25 and Pre-F50) in the rats receiving infusions of vehicle, L-cysteine or D-CSNO were elevated to a similar degree, except the f_R_ values prior to the injection of the 50 μg/kg dose of fentanyl in the rats receiving infusions of vehicle, L-cysteine or D-CSNO. Therefore, these elevations in ventilatory parameters were evidently due to the injections of fentanyl with minimal differences occurring because of the presence of L-cysteine or D-CSNO.

### SNO-L-CYS Infusion Blunts the Negative Effects of Fentanyl on Eupneic Breathing

**FIGURE 9:**
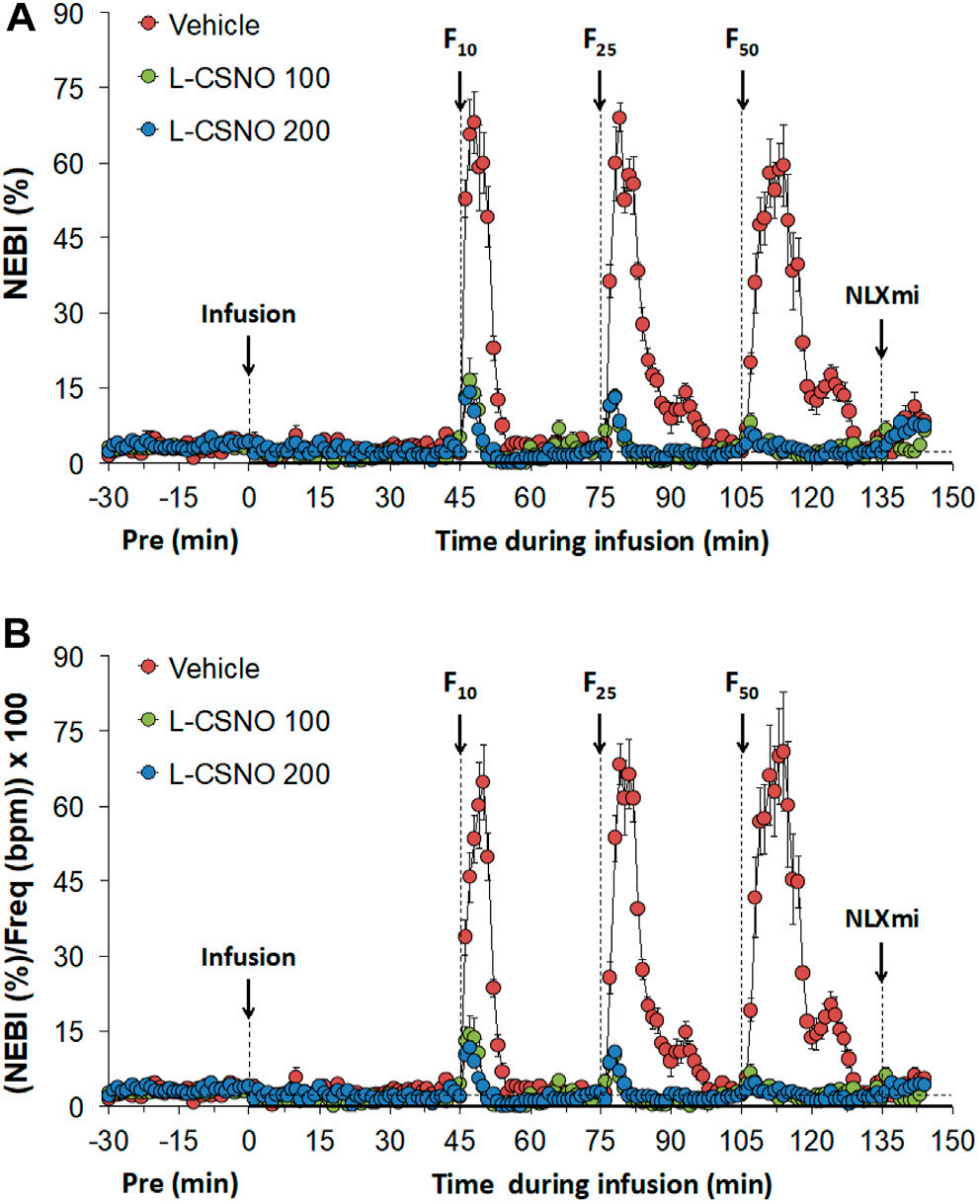
Non-eupneic breathing index (NEBI) **(A)** and NEBI/Frequency of breathing (NEBI/Freq) **(B)** values. The infusion of vehicle (20 μl/min, IV), L-CSNO 100 (S-nitroso-L-cysteine, 100 nmol/kg/min, IV) or L-CSNO 200 (S-nitroso-L-cysteine, 200 nmol/kg/min, IV) commenced at time 0. Bolus intravenous injections of fentanyl at F10 (10 μg/kg, IV), F25 (25 μg/kg, IV), and F50 (50 μg/kg, IV) were given at 45, 75, and 105 min, respectively. A bolus intravenous injection of naloxone methiodide (NLXmi, 1.5 mg/kg, IV) was given at time 135 min. The data are presented as mean ± SEM. There were 8 rats in each group.

The actual NEBI and NEBI/f_R_ values during various stages of the L-CSNO (100 or 200 nmol/kg/min, IV) infusion studies are presented in Figure 9. Figure 9A shows that the baseline NEBI values in all rats were very low, suggesting that most breaths were eupneic in nature. The infusions of vehicle or L-CSNO at 100 or 200 nmol/kg/min minimally affected resting NEBI levels. In vehicle-infused rats, the injection of the 10, 25, and 50 μg/kg doses of fentanyl elicited transient, but substantial, increases in NEBI of 5–10 min in duration. These detrimental effects of fentanyl were virtually absent in rats receiving 100 or 200 nmol/kg/min infusions of L-CSNO. Figure 9B shows that even when corrected for the levels of f_R_, the pattern of effects elicited by fentanyl in rats receiving infusions of vehicle or L-CSNO are qualitatively similar to those shown in Figure 9A. The arithmetic changes in NEBI and NEBI/f_R_ (expressed as differences from the 45 min infusion timepoint) shown in Supplementary Figure S1, confirm that the ability of fentanyl to deleteriously affect NEBI and NEBI/f_R_ are diminished in rats receiving infusions of L-CSNO 100 or L-CSNO 200. Supplementary Figure S2 summarizes the total (cumulative) arithmetic changes in NEBI and NEBI/f_R_ recorded 5 and 30 min after injection of fentanyl (10, 25, and 50 μg/kg doses, IV) in rats receiving continuous infusions of vehicle (20 μl/min, IV), or L-CSNO (100 or 200 nmol/kg/min, IV). The data clearly shows that L-CSNO 100 and 200 dramatically reduce the ability of fentanyl to destabilize breathing. As shown in Supplementary Figures S3, S4 the ability of fentanyl (10, 25, and 50 μg/kg doses, IV) to deleteriously affect NEBI and NEBI/f_R_ was not altered by infusions of L-cysteine (200 nmol/kg/min, IV) or D-CSNO (200 nmol/kg/min, IV).

### Profound Effects of NLXmi in Fentanyl-Injected Rats

**FIGURE 10:**
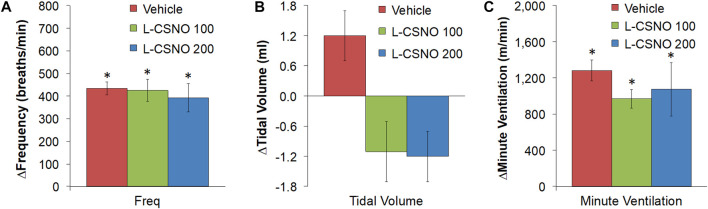
Total arithmetic changes in frequency of breathing **(A)**, tidal volume **(B)**, and minute ventilation **(C)** over the 9-min period following the injection of naloxone methiodide (1.5 mg/kg, IV) in rats receiving infusion of vehicle (20 μl/min, IV), L-CSNO 100 (S-nitroso-L-cysteine, 100 nmol/kg/min, IV), or L-CSNO 200 (S-nitroso-L-cysteine, 200 nmol/kg/min, IV). The data are shown as mean ± SEM. There were 8 rats in each group. *p < 0.05, significant change from Pre values. ^†^ *p* < 0.05, L-CSNO 100 or L-CSNO 200 versus vehicle.

Rather remarkably, the injection of NLXmi (1.5 mg/kg, IV) elicited a prompt and substantial rise in f_R_ in vehicle-infused rats (Figure 1). This effect of NLXmi was strictly related to administration of prior injections of fentanyl, since the injection of NLXmi (1.5 mg/kg, IV) elicited minimal changes in f_R_, V_T_, and V_E_ values in rats receiving the infusions of vehicle, but which received injections of vehicle instead of fentanyl (Supplementary Table S3). As can be seen in Figure 1, the increases in f_R_ elicited by NLXmi in rats receiving the 100 or 200 nmol/kg/min infusions of L-CSNO were similar to those in vehicle-infused rats (Figure 1A). Due to the more dramatic effect of NLXmi on f_R_ than V_T_ (Figure 1B), the net effect of the administration of NLXmi was a marked increase in V_E_ of similar magnitude in all three groups (Figure 1C). The differences in the Pre values (i.e., those prior to any drug administration) for f_R_, V_T_, and V_E_ compared to those values prior to the administration of NLXmi in rats receiving vehicle, L-CSNO 100 and L-CSNO 200 are summarized in Supplementary Table S4. As can be seen, f_R_ values were not changed in rats receiving infusions of vehicle or 100 nmol/kg/min of L-CSNO, whereas f_R_ was elevated in rats receiving the 200 nmol/kg/min infusion of L-CSNO. In addition, V_T_ and V_E_ values were elevated in all three of the groups, but substantially more so in the rats receiving the 200 nmol/kg/min infusion of L-CSNO. The total (cumulative) changes in f_R_, V_T_, and V_E_ elicited by the injection of NLXmi are summarized in Figure 10. The injection of NLXmi elicited pronounced increases in V_E_ (Figure 10C) that were entirely due to the increases in f_R_ (Figure 10A), but not in V_T_ (Figure 10B). Conclusively, it is readily apparent that the NLXmi-induced responses were not modified by the infusions of L-CSNO at either 100 or 200 nmol/kg/min infusions (Figure 10).

The differences in the Pre values (i.e., those prior to any drug administration) for f_R_, V_T_, and V_E_ compared to those values prior to the administration of NLXmi in rats receiving vehicle, L-cysteine or D-CSNO are summarized in Supplementary Table S5. As can be seen, V_T_ and V_E_, but not f_R_ were elevated prior to injection of NLXmi in the three groups (*p* > 0.05 for all between group responses). As seen in Figure 6, the responses were qualitatively similar in all three groups, and the injection of NLXmi elicited prompt and substantial increases in f_R_ that were accompanied by smaller increases in V_T_, which together produced substantial increases in V_E_. Supplementary Figure S5, shows that the total (cumulative) responses recorded during the 9 min period following injection of NLXmi were similar in all groups for f_R_ and V_E_, whereas the total responses for V_T_ were smaller in rats receiving infusions of L-cysteine or D-CSNO than those receiving vehicle infusion. As seen in Supplementary Figure S6, the total changes in NEBI elicited by the injection of NLXmi in rats receiving the 100 or 200 nmol/kg/min infusions of L-CSNO (Supplementary Figure S6A) or the 200 nmol/kg/min infusions of L-cysteine or D-CSNO (Supplementary Figure S6C) were not different from their respective vehicle-infusion groups. However, upon correcting the changes in NEBI for the levels of f_R_, it was evident that the NLXmi-induced changes in NEBI/f_R_ were somewhat greater in rats receiving the 200 nmol/kg/min infusions of L-CSNO (Supplementary Figure S6B) or infusion of L-cysteine (Supplementary Figure S6D).

### L-CSNO, but Not L-Cysteine or D-CSNO Promotes the Antinociceptive Actions of Fentanyl

**FIGURE 11:**
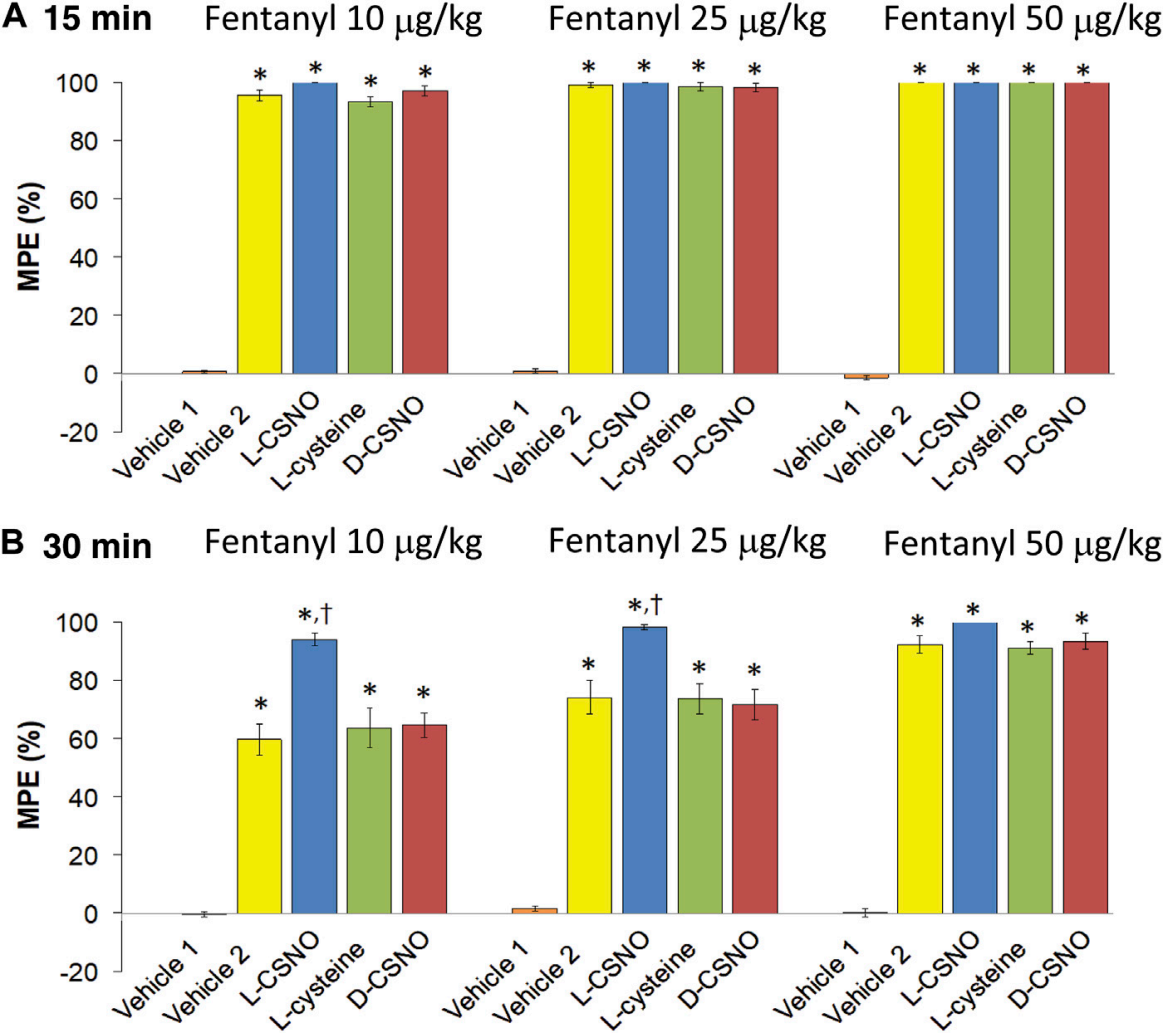
Changes in tail-flick latencies 15 min **(A)** and 30 min **(B)** following injection of fentanyl (10, 25, and 50 μg/kg, IV) expressed as maximal possible effect (MPE, %). Vehicle 1 rats received an infusion of vehicle, three injections of vehicle and then an injection of NLXmi (2.5 mg/kg, IV) 30 min after the third injection of vehicle. Vehicle 2 rats received an infusion of vehicle for 60 min before receiving injections of 10, 25, or 50 μg/kg doses of fentanyl given 30 min apart and then injection of vehicle, 30 min after injection of 50 μg/kg dose of fentanyl. The groups denoted as L-CSNO, L-cysteine or D-CSNO received infusions at 200 nmol/kg/min for 60 min before receiving injections of 10, 25, or 50 μg/kg doses of fentanyl given 30 min apart and an injection of NLXmi (2.5 mg/kg, IV) 30 min after injection of 50 μg/kg dose of fentanyl. The data are presented as mean ± SEM. There were 6 rats in each group. *p < 0.05, significant change from Pre values. ^†^ *p* < 0.05, L-CSNO versus Vehicle 2, L-cysteine and D-CSNO.

**FIGURE 12:**
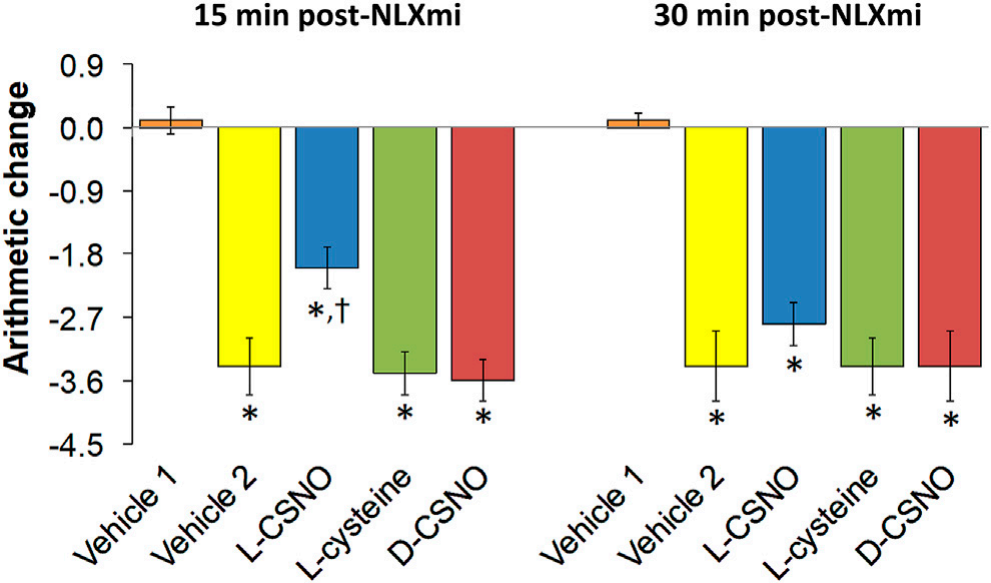
Changes in tail-flick latencies 15 min **(A)** and 30 min **(B)** following injection of naloxone methiodide (NLXmi, 2.5 mg/kg, IV) given 30 min after the injection of vehicle (Vehicle 1) or fentanyl (50 μg/kg, IV). The group denoted Vehicle 1 received an infusion of vehicle, three injections of vehicle and then NLXmi (2.5 mg/kg, IV) 30 min after the third injection of vehicle. The group denoted Vehicle 2 received an infusion of vehicle for 60 min before injection with 10, 25, or 50 μg/kg doses of fentanyl given 30 min apart and then an injection of vehicle 30 min after injection of 50 μg/kg dose of fentanyl. Infusion groups denoted as L-CSNO (S-nitroso-L-cysteine, 200 nmol/kg/min), L-cysteine (200 nmol/kg/min) or D-CSNO (S-nitroso-D-cysteine, 200 nmol/kg/min) were infused for 60 min before receiving injections of 10, 25, or 50 μg/kg doses of fentanyl given 30 min apart and then an injection of NLXmi (2.5 mg/kg, IV) given 30 min after the injection of 50 μg/kg dose of fentanyl. The data are presented as mean ± SEM. There were 6 rats in each group. *p < 0.05, significant change from Pre values. ^†^ *p* < 0.05, L-CSNO versus Vehicle 2, L-cysteine and D-CSNO.

The tail-flick latency (TFL) values of the five treatment groups for the entire experimental protocol are summarized in Supplementary Table S6. The baseline values of each treatment group were similar to one another (*p* > 0.05, for all comparisons). In addition, none of the infusions altered TFL values as recorded at 60 min of infusion, which was immediately preceding injection of the 10 μg/kg dose of fentanyl (*p* > 0.05, for all comparisons based on arithmetic changes). Nonetheless, the arithmetic changes because of all the five treatments are summarized in Supplementary Table S7. The changes in TFL expressed as percent of maximum possible effect (%MPE) are summarized in Figure 11. As seen in Figure 11A, the antinociceptive effects of the 10, 25, and 50 μg/kg doses of fentanyl recorded 15 min after injection were similar in all groups that received fentanyl (i.e., all those except for Vehicle 1, which received injections of vehicle instead). As seen in Figure 11B, the antinociceptive effects of the 10 and 25 μg/kg doses of fentanyl recorded 30 min after injection were clearly less than those recorded at 15 min for all groups except the group of rats receiving infusion of L-CSNO. The antinociceptive effect of the 50 μg/kg dose of fentanyl recorded 30 min after injection was clearly similar to the effect recorded at 15 min for all groups, except Vehicle 1 (Figure 11B). Therefore, it appears that the antinociceptive actions of fentanyl were augmented by L-CSNO at the lower doses of fentanyl, whereas they were not affected by L-cysteine or D-CSNO at either the low or high doses of fentanyl. As summarized in Figure 12, the bolus injection of NLXmi did not affect TFL in rats that received an infusion of vehicle plus three bolus injections of vehicle (Vehicle 1). In contrast, the injection of NLXmi elicited a significant reduction in TFL in the rats that received the injections of fentanyl, namely, the Vehicle 2 rats, L-CSNO rats, L-cysteine rats and D-CSNO rats, as recorded 15 and 30 min post-NLXmi injection. The decrease in TFL at 15 min was less in the L-CSNO rats than in the Vehicle 2, L-cysteine or D-CSNO rats.

## Discussion

### Pharmacological Effects of Fentanyl in Rats Receiving an Infusion of Vehicle

This study demonstrates that consecutive injections of 10, 25, and 50 μg/kg doses of the synthetic opioid, fentanyl (Suzuki and El-Haddad, 2017; Armenian et al., 2018), elicit pronounced biphasic effects on f_R_, V_T_, and V_E_ in unanesthetized male Sprague Dawley rats receiving an infusion of vehicle. The first injection of fentanyl (10 μg/kg) elicited a rapid short-lasting increase in f_R_ that, after return to pre-injection levels (i.e., frequency values before injection of a 10 μg/kg dose fentanyl), began to rise steadily to a plateau level that was 15% higher than the pre-injection levels. This plateau took 15 min to occur. Subsequent injections of 25 and 50 μg/kg doses of fentanyl elicited a transient rise in f_R_ followed by a sustained decrease in f_R_ below pre-injection levels. The changes in V_T_ elicited by fentanyl (10, 25, and 50 μg/kg) consisted of a pronounced initial decrease followed by a period of recovery, with levels being close to pre-injection, and then a gradual rise in V_T_ of about 25% above pre-injection levels, after 15 min. Due to similar changes in f_R_ and V_T_, each dose of fentanyl produced qualitatively similar changes in V_E_ consisting of rapid and large decreases followed by a recovery period and then a rise to about 50% above pre-injection values. While the ability of opioids, such as morphine, to depress breathing and the mechanisms by which this occurs have been extensively studied (Lalley, 2008; Pattinson, 2008; Dahan et al., 2010; Pattinson and Wise, 2016; Baby et al., 2018; Dahan et al., 2018; Bateman et al., 2021; Baby et al., 2021a; Baby et al., 2021b; Ramirez et al., 2021), the sites/mechanisms of action by which fentanyl exerts its cardiorespiratory and antinociceptive effects are less well understood (Laubie et al., 1977; Fone and Wilson, 1986; Mayer et al., 1989; Yamakura et al., 1999; Lalley, 2003; Maegawa and Tonussi, 2003; Griffioen et al., 2004; Mastronicola et al., 2004; Nikolaishvili et al., 2004; Hajiha et al., 2009; Tschirhart et al., 2019; Webster and Rauck, 2021; Ramos-Matos et al., 2022). Mechanisms of action of fentanyl involve activation of μ-ORs in brainstem nuclei controlling cardiorespiratory and nociceptive functions (Laubie et al., 1977; Fone and Wilson, 1986; Lalley, 2003; Griffioen et al., 2004; Nikolaishvili, et al., 2004; Hajiha, et al., 2009; Webster and Rauck, 2021; Ramos-Matos et al., 2022), and in peripheral structures, including the carotid body (Mayer et al., 1989; Henderson et al., 2014; Tschirhart et al., 2019; Ramos-Matos et al., 2022).

Consistent with previous reports (Jenkins et al., 2021; Seckler et al., 2022), each injection of fentanyl in vehicle-infused rats elicited substantial, relatively short-lived increases in occurrence of non-eupneic breaths, as demonstrated by the increases in NEBI and NEBI/f_R_. Based on the known effects of opioids on breathing patterns (Zutler and Holty, 2011; Nagappa et al., 2017), it is likely that most non-eupneic breathing consists of breath-holds (apneas) although other events, such as type 1 and type 2 sighs, may have occurred. There is conflicting data showing that fentanyl has a strong potential for relief of dyspnea/refractory breathlessness in humans (Simon et al., 2013; Campbell, 2017; Higgins et al., 2020). Nonetheless, the ability of fentanyl to produce apneas is well documented (Willette et al., 1987; Yeadon and Kitchen, 1990; Ren et al., 2009; Zhang et al., 2012a,b; Zhuang et al., 2012; Haouzi et al., 2020; Saunders and Levitt, 2020) by mechanisms including activation of μ-OR in 1) ventrolateral medulla (Willette et al., 1987), 2) the Kölliker-Fuse/parabrachial nucleus complex (Saunders and Levitt, 2020), and 3) the NTS (Zhang et al., 2012a; Zhuang et al., 2012). Additionally, there is evidence for μ-OR-mediated activation of pulmonary C-fiber afferents (Zhang et al., 2012a; Zhang et al., 2012b; Zhuang et al., 2012). Moreover, opioids depress ventilatory responses to hypoxic, hypercapnic, and hypoxic-hypercapnic challenges (Berkenbosch, et al., 1997; May et al., 2013a; May et al., 2013b). Accordingly, the ability of fentanyl to depress breathing, and perhaps to elicit non-eupneic breathing, may also involve the suppression of ventilatory adaptations that occur in response to elevations of arterial blood concentrations of protons, and increases in blood CO_2_ and decreases in blood O_2_ (Henderson et al., 2014; Jenkins et al., 2021). These actions of fentanyl may involve activation of μ-ORs in the brainstem, since microinjections of μ-OR agonists into sites in the caudal medullary raphe region inhibit the ventilatory responses to hypercapnic challenges (Zhang et al., 2007), whereas direct application of these agonists in sites in the medullary raphe (Zhang et al., 2009) or commissural NTS (Zhang et al., 2011) reduce ventilatory responses to hypoxic challenges. The ability of the 10, 25, and 50 μg/kg doses of fentanyl in vehicle-infused rats to elicit an increased TFL in unanesthetized rats is consistent with its ability to produce antinociception in rats and other species (Henderson et al., 2014), and to bring pain relief in animals and humans (Hansen et al., 2012; Henderson et al., 2014; Wiffen et al., 2017).

### L-CSNO Markedly Diminishes the Negative Effects of Fentanyl on Breathing

L-CSNO is an endogenous SNO (Myers et al., 1990; Bates et al., 1991; Seckler et al., 2020) with an array of activities (Seth and Stamler, 2011; Marozkina and Gaston, 2012; Stomberski et al., 2019; Marozkina and Gaston, 2020), including modulation of ventilatory control systems (Lipton et al., 2001; Gaston et al., 2006; Gaston et al., 2014; Gaston et al., 2020). The infusion concentrations of L-CSNO (100 and 200 nmol/kg/min) were designed to minimally affect baseline ventilatory parameters to see whether cell-signaling events triggered by L-CSNO prevent fentanyl depression of breathing independently of direct effects of L-CSNO on ventilatory control processes that manifest in changes in baseline ventilation. Our preliminary data found that infusions of SNO-L-CYS at 250 nmol/kg/min elevated V_E_ to plateau levels of +48 ± 6% of baseline values (*n* = 6 rats, *p* < 0.05), which is consistent with our recent evidence that systemic administration of SNO-L-CYS increases V_E_ (Gaston et al., 2020) *via* mechanisms including activation of carotid body chemoafferents (Gaston et al., 2020). This increase in baseline V_E_ would have complicated our interpretation of alterations in the efficacy of fentanyl, and so we used lower concentrations of L-CSNO in the hope that despite minimally changes in baseline values, these concentrations would be efficacious against fentanyl. A key finding was that the ventilatory depressant effects and increases in non-eupneic breathing elicited by fentanyl were markedly reduced in rats receiving L-CSNO at 100 and 200 nmol/kg/min, whereas they were not reduced by 200 nmol/kg/min infusions of D-CSNO, or the parent thiol, L-cysteine. The efficacy of the 100 nmol/kg/min infusion of L-CSNO is noteworthy since this infusion did not alter baseline f_R_, V_T_ or V_E_. It appears that this low concentration of L-CSNO prevented OR-mediated signaling processes independent of baseline changes in ventilation that this SNO can exert.

Our finding that the antinociceptive actions of fentanyl were augmented in rats receiving L-CSNO, taken together with knowledge that SNOs do not directly interact with μ-ORs (Kokkola et al., 2005), support the possibility that L-CSNO does not block ORs, but that it modulates mechanisms and signaling pathways that mediate the respiratory depressant and antinociceptive actions of opioids (Imam et al., 2018; Stein, 2018; Birdsong and Williams, 2020). Processes by which L-CSNO differentially modulates respiratory depressant and antinociceptive actions of fentanyl are not understood, although the inability of L-cysteine or D-CSNO to exert similar effects suggests that the SNO moiety is essential for activity, and its activity relies upon its stereoselective configuration. L-CSNO stereoselectivity in cardiorespiratory systems has been heavily described (Davisson et al., 1996a; Lewis et al., 1996; Davisson et al., 1997a; Ohta et al., 1997; Hoque et al., 1999; Hoque et al., 2000; Lipton et al., 2001; Lewis et al., 2005a; Lewis et al., 2005b, Lewis et al., 2006a; Gaston et al., 2020) and these activities may involve interactions with membrane-bound proteins, including T-type Ca^2+^-channels (Joksovic et al., 2007), glutamate (N-methyl-D-aspartate) ion-channel receptors (Travis and Lewis, 2000), and voltage-gated Kv_1.1_-channels (Gaston et al., 2020). Although stereoselectivity has not been established, L-CSNO activity may involve interactions with Ca^2+^-activated K^+^-channels (Bolotina et al., 1994; George and Shibata, 1995; Forrester et al., 2009; Foster et al., 2009; Lima et al., 2010) and Kv_1.2_- and Kv_1.3_-channels (Garcia et al., 1995). Stereoselectivity of effects of L-CSNO may involve its ability to enter cells *via* L-amino acid transporter (L-AT) systems, which do not transport D-CSNO (Nemoto et al., 2003; Li and Whorton, 2007). Intracellular entry of L-CSNO *via* L-AT would allow it to modulate the activities/functions of signaling pathways (Matsumoto et al., 2003; Burwell et al., 2006; Whalen et al., 2007; Forrester et al., 2009; Broniowska and Hogg, 2010; Iwakiri, 2011; Seth and Stamler, 2011; Piantadosi, 2012; Dejanovic and Schwarz, 2014; Tarasenko, 2015; Lin et al., 2018).

We have reported that NADPH diaphorase histochemistry visualizes S-nitrosylated proteins in peripheral and central structures (Seckler et al., 2020). The finding that morphine administration results in a marked decrease in NADPH diaphorase staining in the brain (Dyuizen et al., 2001; Diuĭzen, et al., 2003; Dyuizen et al., 2004) suggests that opioids may promote the use and/or denitrosylation of S-nitrosylated proteins in the brain, and by analogy in peripheral structures that express NADPH diaphorase staining, such as the carotid body-carotid sinus nerve terminal complex (Atanasova et al., 2020). Establishing higher levels of S-nitrosylated proteins may countermand the ability of opioids to reduce the S-nitrosylation status of cells and this might be an important factor in how L-CSNO infusion diminishes the ability of fentanyl to deleteriously affect breathing. Enhancement of endogenous S-nitrosylation status, by L-CSNO infusion, may enhance the antinociceptive effects of the opioid acting at pain modulation sites.

L-CSNO prevents down regulation/desensitization of G-protein-coupled β-adrenoceptors (Whalen et al., 2000; Whalen et al., 2006), pituitary adenylate-cyclase-activating polypeptide (PACAP) (Whalen et al., 1999), and ligand-gated 5-HT_3_ ion-channel receptors (Owen et al., 2005) by mechanisms that are independent of the NO-cGMP-protein kinase G signaling pathway. β-adrenoceptors are linked mainly to G_s_ proteins and to a lesser degree, G_i_ proteins (Frielle et al., 1989; Rubenstein et al., 1991; Rockman et al., 2002; Mori et al., 2020), whereas PACAP receptors are linked mainly to G_s_, but also to G_q_ and G_i_/G_o_ proteins (Van Rampelbergh et al., 1997; Martin et al., 2005). *μ*-, *δ*-, and *κ*-ORs couple to G_i_/_o_-proteins (Roerig et al., 1992; Laugwitz et al., 1993; Prather et al., 1995; Law et al., 2000) with signaling effects including, inhibition of adenylate cyclase (Sharma et al., 1977; Law et al., 2000), inhibition of Ca^2+^ channels (Hescheler et al., 1987), activation of 1) G protein-coupled inwardly-rectifying K^+^ channels (North et al., 1987; Henry et al., 1995), and 2) mitogen-activated protein kinase (Fukuda et al., 1996), and stimulation of phospholipase C (Spencer et al., 1997). S-nitrosylation signaling events triggered by L-CSNO directly modulate activities of signaling proteins (George and Shibata, 1995; Gaston et al., 2003; Gonzalez et al., 2009; Lima et al., 2010; Kayki-Mutlu and Koch, 2021). We are examining the literature about S-nitrosylation targets for L-CSNO (Matsumoto et al., 2003; Burwell et al., 2006; Forrester et al., 2009; Foster et al., 2009; Lima et al., 2010; Numajiri et al., 2011; Seth and Stamler, 2011; Haldar and Stamler, 2013; Shao et al., 2016; Stomberski et al., 2019) to design studies to better understand molecular mechanisms by which L-CSNO affects the actions of fentanyl.

### L-CSNO Augments Fentanyl-Induced Antinociception

SNOs modulate G protein-coupled receptor signaling in a receptor-specific and reversible manner (Whalen et al., 1999; Whalen et al., 2007; Kokkola et al., 2005; Nozik-Grayck et al., 2006), whereas neither L-CSNO nor S-nitroso-L-glutathione directly interact with μ-ORs (Kokkola et al., 2005). As such, the ability of L-CSNO infusion to augment the duration of fentanyl-induced analgesia (particularly evident for the 10 and 25 μg/kg doses) may involve modulation of the signaling pathways controlling pain perception that are activated by fentanyl (Hansen et al., 2012; Henderson et al., 2014; Wiffen et al., 2017). The mechanisms by which L-CSNO infusion promotes fentanyl analgesia may involve the release NO because of evidence that peripheral antinociceptive actions of fentanyl are mediated *via* the NO/cyclic-GMP/protein kinase G pathway (Maegawa and Tonussi, 2003), and that opioids, such as morphine, induce pain relief *via* the NO/cyclic-GMP/protein kinase G pathway (Ferreira et al., 1991; Leánez et al., 2009; Cunha et al., 2010; Cury et al., 2011; Gomes et al., 2020). There is also substantial evidence that NO is a cGMP/protein kinase G-dependent pronociceptive agent that drives nociceptive hyper-sensitivity (Tegeder et al., 2011), and that NOS inhibitors reduce nociceptive behaviors (Hao and Xu, 1996; Aley et al., 1998; Levy and Zochodne, 1998; Chen and Levine, 1999). The ability of L-CSNO to augment fentanyl antinociception may involve L-CSNO activation and/or S-nitrosylation of functional proteins. S-nitrosylation of nociceptive signaling proteins include 1) transient receptor potential channels, 2) voltage-gated channels, 3) G-protein-coupled receptors, 4) glutamate receptors, 5) redoxins, and 6) pro-inflammatory enzymes (Tegeder et al., 2011). S-nitrosylation of these proteins requires a permissive redox state of sulfur atoms, and includes changes of ion channel gating properties, modulation of membrane fusion and fission processes that regulate insertion/removal of receptors/ion-channels into plasma membranes, and alteration of protein ubiquitination status and protein degradation. The roles of NO and SNO-dependent signaling events in the ability of L-CSNO to augment fentanyl-induced antinociception awaits further study.

### Novel Effects of NLXmi in Fentanyl-Treated Rats

The novel finding that NLXmi elicited a pronounced increase in f_R_, whereas it decreased V_T_ in vehicle-infused rats that received injections of fentanyl (10, 25, and 50 μg/kg), but not injections of vehicle, raises important questions. First, injections of fentanyl elicited excitatory and inhibitory effects on breathing *via* peripheral and/or central pathways that sub-serve the opposing actions of fentanyl. While the adverse effects of opioids on breathing have been extensively characterized (Dahan et al., 2010; Dahan et al., 2018; Gaston et al., 2020), there is also evidence that lower doses of opioids, including fentanyl, morphine, methadone, dermorphin, and other *μ-* and *δ*-OR agonists, stimulate ventilation (Metcalfe et al., 1980; Hassen et al., 1982; Haddad et al., 1984; Hurle et al., 1985; Schaeffer and Haddad, 1985; Szeto et al., 1988; Paakkari et al., 1990; Szeto et al., 1991; Paakkari et al., 1993; Henderson et al., 2013; Henderson et al., 2014). Respiratory stimulation occurred upon many sites/methods of administration (Metcalfe et al., 1980; Paakkari et al., 1993; Henderson et al., 2013; Henderson et al., 2014), including NTS (Hassen et al., 1982), ventral medullary and dorsal pontine surfaces (Hurle et al., 1985), lateral ventricles (Paakkari et al., 1990; Paakkari et al., 1993) and fourth ventricle (Haddad et al., 1984; Schaeffer and Haddad, 1985) of the brain, and fetal inferior vena cava (Szeto et al., 1988; Szeto et al., 1991).

NLXmi increased f_R_ in fentanyl-injected rats at 30 min post-fentanyl 50 μg/kg injection when resting f_R_ was back to baseline. An explanation for the effects of NLXmi in fentanyl-treated rats is that repeated injections of fentanyl activate opposing pathways, one that promotes and one that depresses breathing, and that these opposing drives result in a normal f_R_. The inhibitory pathway that controls f_R_ may involve NLXmi-sensitive peripheral OR signaling processes, and the effects of this excitatory pathway expressed after injection of NLXmi on f_R_ may be due to non-OR-driven pathways in the periphery and/or to central OR and/or non-OR-driven systems. In contrast to f_R_, V_T_ was elevated at the time NLXmi was given to fentanyl-injected rats receiving vehicle infusion. The decrease in V_T_ elicited by NLXmi suggests that tonically active peripheral OR-driven systems were involved in the increases in V_T_ after injections of fentanyl. It is difficult to speculate on peripheral mechanisms involved in responses to NLXmi, but differential effects occur *via* OR signaling cascades in structures without blood-brain barriers, including the area postrema (Guan et al., 1999), anteroventral region of the third ventricle (Feuerstein et al., 1985), subfornical organ (Atwah and Kuhar, 1977), and carotid bodies (McQueen and Ribeiro, 1980; Zimpfer et al., 1983; Kirby and McQueen, 1986; Mayer et al., 1989; Sarton et al., 1999), noting that sex differences in morphine-induced ventilatory depression reside in peripheral chemoreflex loops (Sarton et al., 1999). Despite the effects of L-CSNO on immediate changes in f_R_, V_T_, and V_E_ elicited by fentanyl, the ventilatory responses elicited by NLXmi in rats receiving L-CSNO infusion were similar to those in vehicle-infused rats. Whatever mechanisms are involved in recruiting fentanyl-induced tonically active excitatory/inhibitory ventilatory control pathways, they may not be subject to control by L-CSNO or downstream events, including S-nitrosylation (Lipton, et al., 1993; Matsumoto et al., 2003; Forrester, et al., 2009). Despite a robust increase in f_R_, NLXmi elicited minor changes in NEBI and NEBI/f_R_ in fentanyl-injected rats receiving vehicle or L-CSNO. As such, fentanyl did not initiate NLXmi-sensitive control processes that perturb eupneic breathing.

## Study Limitations

The main finding that continuous infusion of L-CSNO in unanesthetized adult male Sprague Dawley rats diminishes the detrimental effects fentanyl has on breathing should be confirmed in female rats. There is a major gap in understanding of sex differences in 1) ventilatory control processes and the roles of SNOs in these processes (Palmer et al., 2013a; Getsy et al., 2021), 2) S-nitrosylation events (Brown-Steinke et al., 2010; Shao et al., 2016; Casin and Kohr, 2020), and 3) ventilatory responses to opioids in naïve rats or those subjected to interventions such as, inhibition of NOS (Dahan et al., 1998; Sarton et al., 1999; Hosseini et al., 2011; Gaulden et al., 2021). A more circumspect set of studies in which a single higher dose of fentanyl (e.g., 75 μg/kg) is injected into rats receiving an infusion of L-CSNO may provide more straight-forward results to allow additional interventions (e.g., injection of enzyme inhibitors) aimed at elucidating mechanisms by which L-CSNO has profound effects on the ability of fentanyl to deleteriously affect breathing and NEBI. Studies using centrally active μ-OR antagonists (Dahan et al., 2010) or δ-OR antagonists (Young et al., 2013) will help to define the roles of peripheral and central OR systems associated with the effects of NLXmi in fentanyl-injected rats. Moreover, it would seem essential to establish the potency and efficacy of L-CSNO in dose-response studies to see whether even lower infusion concentrations of L-CSNO will be efficacious against fentanyl. As mentioned above, a limitation of this study is the lack of understanding of molecular mechanisms by which L-CSNO modulates the ventilatory and antinociceptive actions of fentanyl. Therefore, we are currently determining whether systemic injections of fentanyl generate SNOs in central or peripheral structures in rats using capacitative sensor technology (Seckler et al., 2017), and whether infusions of fentanyl change the S-nitrosylation status of central/peripheral structures by NADPH diaphorase histochemistry, which visualizes S-nitrosylated proteins (Seckler et al., 2020).

## Conclusion

This study reports that the deleterious effects of fentanyl on f_R_, V_T_, V_E_, and ventilatory, stability, as defined by the increase in NEBI, were diminished in unanesthetized rats receiving a continuous intravenous infusion of L-CSNO, but not in those receiving continuous intravenous infusion of L-cysteine or D-CSNO. Additionally, the antinociceptive actions of fentanyl were augmented by L-CSNO, but not L-cysteine or D-CSNO. As such, we conclude that the ability of L-CSNO to exert these therapeutically relevant responses against the detrimental effects of fentanyl may involve the activation of stereoselective signal transduction processes, which may include the activation of membrane signaling proteins (Travis and Lewis, 2000; Joksovic et al., 2007; Gaston et al., 2020) and/or intracellular entry *via* the L-amino acid transporter (Li et al., 2000; Nemoto et al., 2003; Li and Whorton, 2007), which allows for modulation of intracellular signaling pathways. Taking into consideration the findings that the peripherally-restricted μ-OR antagonist, NLXmi, substantially reduces ventilatory and antinociceptive responses elicited by fentanyl (Henderson et al., 2014), we conclude that L-CSNO modulation of the effects of fentanyl involve interactions with both peripheral and central μ-OR signaling pathways.

## Data Availability

The raw data supporting the conclusion of this article will be made available by the authors, without undue reservation.

## Abbreviations

SNO, S-nitrosothiol; L-CSNO, S-nitroso-L-cysteine; D-CSNO, S-nitroso-D-cysteine; f_R_, frequency of breathing; V_T_, tidal volume; V_E_, minute ventilation; TFL, tail-flick withdrawal latency; L-NAME, N^G^-nitro-L-arginine methyl ester; NTS, nucleus tractus solitarius; NO, nitric oxide; NOS, nitric oxide synthase; nNOS, neuronal nitric oxide synthase.
