# Supplementary material for: S-Nitroso-L-Cysteine Stereoselectively Blunts the Deleterious Effects of Fentanyl on Breathing While Augmenting Antinociception in Freely-Moving Rats
Source: Front Pharmacol. 2022 May 26;13:892307. doi: 10.3389/fphar.2022.892307 (PMC9199495; doi:10.3389/fphar.2022.892307)
Supplement: Supplementary file 1 [file DataSheet1.docx]

**Supplemental File**

**S-Nitroso-L-Cysteine Stereoselectively Blunts the Deleterious Effects of Fentanyl on Breathing While Augmenting Antinociception in Freely-Moving Rats**

Paulina M. Getsy,^1,^* Santhosh M. Baby,^2,†^ Ryan B. Gruber,^2^ Ben Gaston,^3^ Tristan H. J. Lewis,^1^

Alan Grossfield,^4^ James M. Seckler,^5^ Yee-Hsee Hsieh,^6^ James N. Bates,^7^ Stephen J. Lewis^1,8,9^

*^1^Department of Pediatrics, Case Western Reserve University, Cleveland, OH, USA*

*^2^Galleon Pharmaceuticals, Inc., 213 Witmer Road, Horsham, PA, USA*

*^3^Herman B Wells Center for Pediatric Research, Indiana University School of Medicine,*

*Indianapolis, IN, 46202, USA*

*^4^Department of Biochemistry and Biophysics, University of Rochester Medical Center,*

*Rochester, NY 14642, USA*

*^56^Department of Biomedical Engineering, Case Western Reserve University,*

*Cleveland, Ohio 44106, USA*

*^6^Division of Pulmonary, Critical Care and Sleep Medicine, Case Western Reserve University, Cleveland, OH, United State*

*^7^Department of Anesthesia, University of Iowa, Iowa City, IA, USA*

*^8^Departments of Pharmacology, Case Western Reserve University, Cleveland, OH, USA*

*^9^Functional Electrical Stimulation Center, Case Western Reserve University, Cleveland, OH, USA*

**Short Title:** S-nitroso-L-cysteine and breathing

**^†^Current address:** Santhosh M. Baby, Translational Sciences Treatment Discovery, Galvani Bioelectronics, Inc., 1250 S Collegeville Rd., Collegeville, Pennsylvania 19426.

Email: santhosh.m.baby@galvani.bio

***Corresponding Author:** Paulina M. Getsy, PhD. Department of Pediatrics, Division of Pulmonology, Allergy and Immunology, Department of Pharmacology School of Medicine, Biomedical Research Building, Room 831, Case Western Reserve University, 10900 Euclid Avenue, Cleveland, OH 44106-4984. Phone: 440-488-5079. Email: pxg55@case.edu

**Supplementary Table S1**

Description of the five treatment groups used in the tail-flick latency experiments

| **Group** | **Weight (g)** | **Infusion** |  | **Bolus injections** | | |  | **Vehicle/NLXmi** |
| --- | --- | --- | --- | --- | --- | --- | --- | --- |
| Group 1 – *Vehicle 1* | 320 ± 3 | 20 μL/min |  | Vehicle | Vehicle | Vehicle |  | NLXmi, 2.5 mg/kg, |
| Group 2 – *Vehicle 2* | 319 ± 3 | 20 μL/min |  | F10 | F25 | F50 |  | Vehicle, 100 μL/kg |
| Group 3 –  *L-CSNO* | 317 ± 3 | 200 nmol/kg/min |  | F10 | F25 | F50 |  | NLXmi, 2.5 mg/kg |
| Group 4 –  *L-Cysteine* | 318 ± 3 | 200 nmol/kg/min |  | F10 | F25 | F50 |  | NLXmi, 2.5 mg/kg |
| Group 5–  *D-CSNO* | 322 ± 3 | 200 nmol/kg/min |  | F10 | F25 | F50 |  | NLXmi, 2.5 mg/kg |

L-CSNO, S-nitroso-L-cysteine. D-CSNO, S-nitroso-D-cysteine. NLXmi, naloxone methiodide. F10, F25 and F50 represent intravenous injection of 10, 25 or 50 μg/kg, respectively. There were 6 rats in each group. The body weights of the five groups of rats (mean ± SEM) were similar to one another (P > 0.05, for all comparisons).

**Detailed description of Statistical Approaches**

The recorded data (1 min bins), derived parameters, and response areas (cumulative percent changes from designated pre-values) were taken for statistical analyses. The pre-drug 1 min bins excluded the occasional marked deviations from resting due to erratic movements of the rats such as scratching. The exclusions ensured accurate determinations of baseline parameters. The data are presented as mean ± SEM. All of the data were analyzed by one-way or two-way analysis of variance followed by Student’s modified t test with Bonferroni corrections for multiple comparisons between means employing the modified error mean square term (EMS) from the ANOVA (Wallenstein et al., 1980). The modified t-statistic is t = (mean group 1- mean group 2)/[s x (1/n1 + 1/n2)^1/2^] where s^2^ = the mean square within groups term from the ANOVA (the square root is taken for the modified t-statistic formula) and n1 and n2 are the numbers of the rats in each group. Based on an elementary inequality called Bonferroni’s inequality, a conservative critical value for the modified t-statistics were obtained from tables of the t-distribution using a significance level of P/m, where m is the number of comparisons between groups to be performed. The degrees of freedom are those for the mean square for the within group variation from the ANOVA table. In most cases, the critical Bonferroni value cannot simply be obtained from conventional tables of the t- distribution but may be approximated from widely available tables of the normal curve by t* = z + (z + z3)/4n, where n is the degrees of freedom and z is the critical normal curve value for P/m (Winer, 1971). As demonstrated clearly by Wallenstein et al (1980), the Bonferroni procedure is recommended for general use since it (a) is the easiest to apply, (b) has the widest range of applications, (c) gives critical values lower than those of other procedures if the investigator is able to limit the number of comparisons, and (d) gives critical values that will be only slightly larger than those of other procedures if many comparisons are made. A value of P < 0.05 was taken as the initial level of statistical significance (Winer, 1971; Wallenstein et al., 1980).

**References for text**

Wallenstein, S., Zucker, C.L., and Fleiss, J.L. (1980). Some statistical methods useful in circulation research. *Circ. Res*. 47, 1-9. doi: 10.1161/01.res.47.1.1.

Winer, B.J. (1971). Statistical principles of experimental design. McGraw-Hill Book Co., pp 752-809.

**Supplementary Table S2**

Baseline ventilatory parameters at the beginning of the experiments (Pre) and prior to each injection of fentanyl (F10, F25 and F50) in the L-cysteine and D-CSNO infusion studies

| **Parameter** |  | **Group** |  | **Pre** |  | **Pre-F10** |  | **Pre-F25** |  | **Pre-F50** |
| --- | --- | --- | --- | --- | --- | --- | --- | --- | --- | --- |
| Frequency, breaths/min |  | Vehicle |  | 107 ± 3 |  | 102 ± 4 |  | 127 ± 4* |  | 118 ± 7 |
|  |  | L-Cysteine |  | 108 ± 3 |  | 104 ± 4 |  | 126 ± 3* |  | 121 ± 7 |
|  |  | D-CSNO |  | 107 ± 3 |  | 105 ± 4 |  | 127 ± 3* |  | 120 ± 6 |
| Tidal Volume, ml |  | Vehicle |  | 2.18 ± 0.17 |  | 2.18 ± 0.09 |  | 2.83 ± 0.09* |  | 2.83 ± 0.13* |
|  |  | L-Cysteine |  | 2.23 ± 0.08 |  | 2.25 ± 0.10 |  | 2.90 ± 0.13* |  | 2.87 ± 0.15* |
|  |  | D-CSNO |  | 2.20 ± 0.02 |  | 2.21 ± 0.05 |  | 2.77 ± 0.07* |  | 2.78 ± 0.07* |
| Minute Ventilation, ml/min |  | Vehicle |  | 233 ± 9 |  | 222 ± 12 |  | 359 ± 15* |  | 332 ± 19* |
|  |  | L-Cysteine |  | 239 ± 12 |  | 234 ± 14 |  | 367 ± 20* |  | 349 ± 25* |
|  |  | D-CSNO |  | 236 ± 8 |  | 229 ± 11 |  | 350 ± 11* |  | 331 ± 9* |
| NEBI, % of epoch |  | Vehicle |  | 3.5 ± 0.3 |  | 5.2 ± 0.3* |  | 5.1 ± 0.9 |  | 7.7 ± 0.7 |
|  |  | L-Cysteine |  | 4.1 ± 0.4 |  | 5.4 ± 0.3 |  | 6.2 ± 0.9* |  | 6.8 ± 0.4* |
|  |  | D-CSNO |  | 3.9 ± 0.3 |  | 4.9 ± 0.4 |  | 5.7 ± 0.2* |  | 6.5 ± 0.2* |
| ((NEBI, %)/Frequency, bpm)) x 100 |  | Vehicle |  | 3.3 ± 0.4 |  | 5.1 ± 0.3 |  | 4.0 ± 0.8 |  | 6.6 ± 0.6* |
|  |  | L-Cysteine |  | 3.8 ± 0.3 |  | 3.2 ± 0.4 |  | 5.2 ± 0.6 |  | 2.5 ± 0.6 |
|  |  | D-CSNO |  | 3.6 ± 0.4 |  | 4.5 ± 0.4 |  | 2.7 ± 0.7 |  | 2.6 ± 0.4 |

Frequency, frequency of breathing. NEBI, non-eupneic breathing index. bpm, breaths per minute. D-CSNO, S-nitroso-D-cysteine. F10, F25 and F50, fentanyl at intravenous doses of 10, 25 and 50 μg/kg, respectively. The data are presented as mean ± SEM. There were 8 rats in each group. *P < 0.05, significant change from Pre values.

**Supplementary Figure S1**

**A.**

**B.**

**Supplementary Figure S1.** Arithmetic changes in non-eupneic breathing index (NEBI) (**Panel A**) and NEBI/frequency of breathing (NEBI/Freq) (**Panel B**) elicited by injections of fentanyl at F10 (10 μg/kg, IV), F25 (25 μg/kg, IV) and F50 (50 μg/kg, IV) in rats receiving an intravenous infusion of vehicle (20 μL/min, IV), S-nitroso-L-cysteine (L-CSNO) at 100 nmol/kg/min, (L-CSNO 100) or 200 nmol/kg/min (L-CSNO 200) commenced at time 0. An injection of naloxone methiodide (NLXmi, 1.5 mg/kg, IV) was given at time 90 min. The data are presented as mean ± SEM. There were 8 rats in each group.

**Supplementary Figure S2**

**30 min sums**

**5 min sums**

**A.**

**B.**

**D.**

**C.**

**Supplementary Figure S2.** Total (cumulative) arithmetic changes in non-eupneic breathing index (NEBI) recorded 5 min (**Panel A**) or 30 min (**Panel B**) after injection of fentanyl at F10 (10 μg/kg, IV), F25 (25 μg/kg, IV) and F50 (50 μg/kg, IV) in rats receiving continuous intravenous infusion of vehicle (20 μL/min, IV), L-CSNO 100 (S-nitroso-L-cysteine, 100 nmol/kg/min, IV) or L-CSNO 200 (200 nmol/kg/min, IV). The total changes in NEBI/frequency of breathing (NEBI/Freq) during the 5 min or 30 min recording periods are shown in **Panels C** and **D**, respectively. The data are presented as mean ± SEM. There were 8 rats in each group. *P < 0.05, significant change from Pre values. ^†^P < 0.05, L-CSNO 100 or L-CSNO 200 versus vehicle.

**Supplementary Figure S3**

**C.**

**A.**

**B.**

**D.**

**Supplementary Figure S3.** Values of non-eupneic breathing index (NEBI) (**Panel A**) and NEBI/frequency of breathing (NEBI/Freq) (**Panel B**) before and following infusion of vehicle (20 μL/min, IV), L-cysteine (200 nmol/kg/min, IV) or S-nitroso-D-cysteine (D-CSNO, 200 nmol/kg/min, IV) that commenced at time 0. Injections of fentanyl at F10 (10 μg/kg, IV), F25 (25 μg/kg, IV), and F50 (50 μg/kg, IV) were given 30 min apart. An injection of naloxone methiodide (NLXmi, 1.5 mg/kg, IV) was given at time 135 min. The arithmetic changes from Pre values in NEBI (**Panel C**) and NEBI/Freq (**Panel D**) are also shown. The data are presented as mean ± SEM. There were 8 rats in each group.

**Supplementary Figure S4**

**30 min sum**

**5 min sum**

**A.**

**C.**

**D.**

**B.**

**Supplementary Figure S4.** Total (cumulative) changes in non-eupneic breathing index (NEBI) and NEBI/Frequency of breathing (NEBI/Freq) elicited by injections of fentanyl at F10 (10 μg/kg, IV), F25 (25 μg/kg, IV), and F50 (50 μg/kg, IV) during the 5 min (**Panels A** **and B**, respectively) or 30 min (**Panels C** **and D**, respectively) periods following these injections in rats receiving continuous infusion of vehicle (20 μL/min, IV), L-cysteine (200 nmol/kg/min, IV) or S-nitroso-D-cysteine (D-CSNO, 200 nmol/kg/min, IV. The data are presented as mean ± SEM. There were 8 rats in each group. *P < 0.05, significant change from Pre values.

**Supplementary Table S3**

Changes in frequency of breathing (f_R_), tidal volume (V_T_) and minute ventilation (V_E_) elicited by bolus injections of vehicle and naloxone methiodide in rats that were receiving a continuous infusion of vehicle

| **Experimental Stage** |  | **fr, breaths/min** |  | **Vt, ml** |  | **Ve, ml/min** |
| --- | --- | --- | --- | --- | --- | --- |
| **Baseline** |  | 111 ± 2 |  | 2.20 ± 0.04 |  | 244 ± 8 |
| **45 min post-vehicle infusion (PV)** |  | 111 ± 2 |  | 2.21 ± 0.05 |  | 245 ± 10 |
| %change from Baseline |  | -0.3 ± 0.7 |  | +0.6 ± 1.0 |  | +0.3 ± 1.0 |
| **Vehicle injection 1 values** |  |  |  |  |  |  |
| 15 min value |  | 111 ± 2 |  | 2.22 ± 0.05 |  | 246 ± 10 |
| 30 min value |  | 111 ± 2 |  | 2.18 ± 0.04 |  | 241 ± 7 |
| %change from Pre – 15 min |  | +0.1 ± 1.1 |  | +0.5 ± 0.8 |  | +0.6 ± 1.6 |
| %change from Pre – 30 min |  | -0.1 ± 0.7 |  | -1.2 ± 0.8 |  | -1.3 ± 1.0 |
| **Vehicle injection 2 values** |  |  |  |  |  |  |
| 15 min value |  | 112 ± 3 |  | 2.23 ± 0.04 |  | 250 ± 10 |
| 30 min value |  | 111 ± 2 |  | 2.23 ± 0.05 |  | 249 ± 9 |
| %change from PV – 15 min |  | +0.9 ± 1.4 |  | +1.1 ± 1.4 |  | 2.0 ± 1.2 |
| %change from PV – 30 min |  | -0.1 ± 0.7 |  | +0.8 ± 1.5 |  | +0.8 ± 1.8 |
| **Vehicle injection 3 values** |  |  |  |  |  |  |
| 15 min value |  | 113 ± 2 |  | 2.24 ± 0.05 |  | 253 ± 8 |
| 30 min value |  | 111 ± 2 |  | 2.23 ± 0.05 |  | 248 ± 8 |
| %change from PV – 15 min |  | +2.1 ± 1.4 |  | +1.4 ± 1.4 |  | +3.6 ± 2.2 |
| %change from PV – 30 min |  | +0.2 ± 0.6 |  | +1.2 ± 1.9 |  | +1.4 ± 1.9 |
| **NLXmi responses** |  |  |  |  |  |  |
| Maximal response |  | 112 ± 3 |  | 2.24 ± 0.06 |  | 252 ± 9 |
| %change from injection 3 value at 30 min |  | +1.4 ± 1.1 |  | +0.1 ± 1.6 |  | +1.5 ± 1.8 |

Body weights of the rats were 317 ± 2 gram. fr, frequency of breathing; Vt, tidal volume; Ve, minute ventilation; NLXmi, naloxone methiodide (1.5 mg/kg, IV). The data are presented as mean ± SEM. There were 8 rats in each group. There were no significant changes in any parameter as a result of the infusion of vehicle, the injections of vehicle or the injection of NLXmi (P > 0.05, for all comparisons).

**Supplementary Table S4**

Differences in parameters between those prior to any drug administration (Pre) and those immediately before administration of naloxone methiodide (Pre-NLXmi)

| **Parameter** |  | **Group** |  | **Pre** |  | **Pre-NLXmi** |  | **Delta** |
| --- | --- | --- | --- | --- | --- | --- | --- | --- |
| Frequency, breaths/min |  | Vehicle |  | 108 ± 3 |  | 108 ± 4 |  | +0.4 ± 1.6 |
|  |  | L-CSNO 100 |  | 108 ± 2 |  | 109 ± 6 |  | +0.5 ± 4.5 |
|  |  | L-CSNO 200 |  | 106 ± 2 |  | 117 ± 2 |  | +11 ± 3* |
| Tidal Volume, ml |  | Vehicle |  | 2.12 ± 0.03 |  | 2.57 ± 0.14 |  | +0.5 ± 0.1* |
|  |  | L-CSNO 100 |  | 2.13 ± 0.03 |  | 2.73 ± 0.10 |  | +0.6 ± 0.1* |
|  |  | L-CSNO 200 |  | 2.17 ± 0.02 |  | 3.22 ± 0.06 |  | +1.1 ± 0.1* |
| Minute Ventilation, ml/min |  | Vehicle |  | 229 ± 6 |  | 280 ± 19 |  | +51 ± 16* |
|  |  | L-CSNO 100 |  | 230 ± 6 |  | 300 ± 27 |  | +70 ± 22* |
|  |  | L-CSNO 200 |  | 231 ± 6 |  | 376 ± 8 |  | +145 ± 10* |
| NEBI, % of epoch |  | Vehicle |  | 3.3 ± 0.4 |  | 4.4 ± 0.9 |  | +1.2 ± 1.0 |
|  |  | L-CSNO 100 |  | 3.1 ± 0.2 |  | 2.7 ± 0.2 |  | -0.4 ± 0.2 |
|  |  | L-CSNO 200 |  | 3.5 ± 0.2 |  | 2.4 ± 0.4 |  | -1.1 ± 0.4* |
| ((NEBI, %)/Frequency, bpm)) x 100 |  | Vehicle |  | 3.0 ± 0.3 |  | 4.0 ± 0.8 |  | +1.1 ± 1.0 |
|  |  | L-CSNO 100 |  | 2.9 ± 0.1 |  | 2.5 ± 0.1 |  | -0.5 ± 0.2* |
|  |  | L-CSNO 200 |  | 3.4 ± 0.2 |  | 2.1 ± 0.3 |  | -1.2 ± 0.4* |

L-CSNO 100 (S-nitroso-L-cysteine at 100 nmol/kg/min, IV). L-CSNO 200 (S-nitroso-L-cysteine at 200 nmol/kg/min, IV). Frequency, frequency of breathing. NEBI, non-eupneic breathing index. bpm, breaths per minute. NLXmi, naloxone methiodide (1.5 mg/kg, IV). The data are presented as mean ± SEM. There were 8 rats in each group. *P < 0.05, significant difference from Pre values.

**Supplementary Table S5**

Differences in parameters between those prior to any drug administration (Pre) and those immediately before administration of naloxone methiodide (Pre-NLXmi)

| **Parameter** |  | **Group** |  | **Pre** |  | **Pre-NLXmi** |  | **Delta** |
| --- | --- | --- | --- | --- | --- | --- | --- | --- |
| Frequency, breaths/min |  | Vehicle |  | 107 ± 3 |  | 107 ± 3 |  | -0.5 ± 3.0 |
|  |  | L-Cysteine |  | 108 ± 3 |  | 104 ± 3 |  | -3.8 ± 1.4* |
|  |  | D-CSNO |  | 107 ± 3 |  | 106 ± 3 |  | -1.3 ± 1.0 |
| Tidal Volume, ml |  | Vehicle |  | 2.18 ± 0.17 |  | 2.51 ± 0.13 |  | +0.33 ± 0.14* |
|  |  | L-Cysteine |  | 2.23 ± 0.08 |  | 2.80 ± 0.18 |  | +0.57 ± 0.12* |
|  |  | D-CSNO |  | 2.20 ± 0.02 |  | 2.68 ± 0.12 |  | +0.45 ± 0.13* |
| Minute Ventilation, ml/min |  | Vehicle |  | 233 ± 9 |  | 266 ± 10 |  | +33 ± 14* |
|  |  | L-Cysteine |  | 239 ± 12 |  | 290 ± 21 |  | +51 ± 13* |
|  |  | D-CSNO |  | 236 ± 8 |  | 283 ± 18 |  | +46 ± 16* |
| NEBI, % of epoch |  | Vehicle |  | 3.5 ± 0.3 |  | 4.5 ± 0.9 |  | +1.0 ± 1.0 |
|  |  | L-Cysteine |  | 4.1 ± 0.4 |  | 4.6 ± 0.7 |  | +0.5 ± 1.1 |
|  |  | D-CSNO |  | 3.9 ± 0.3 |  | 4.4 ± 0.8 |  | +0.5 ± 0.9 |
| ((NEBI, %)/Frequency, bpm)) x 100 |  | Vehicle |  | 3.3 ± 0.4 |  | 4.3 ± 0.9 |  | +1.8 ± 0.5* |
|  |  | L-Cysteine |  | 3.8 ± 0.3 |  | 4.6 ± 0.8 |  | +0.8 ± 0.9 |
|  |  | D-CSNO |  | 3.6 ± 0.4 |  | 3.7 ± 0.7 |  | +0.1 ± 0.7 |

Frequency, frequency of breathing. NEBI, non-eupneic breathing index. bpm, breaths per minute. L-Cysteine (200 nmol/kg/min, IV). S-nitroso-D-cysteine (D-CSNO, 200 nmol/kg/min, IV). NLXmi, naloxone methiodide (1.5 mg/kg, IV). The data are presented as mean ± SEM. There were 8 rats in each group. *P < 0.05, significant difference from Pre values.

**Supplementary Figure S5**

**A.**

**B.**

**C.**

**Supplementary Figure S5.** Total arithmetic changes in frequency of breathing (**Panel A**), tidal volume (**Panel B**), and minute ventilation (**Panel C**), recorded over the nine-minute period after injection of naloxone methiodide (1.5 mg/kg, IV) in the groups that were receiving vehicle (20 μL/min, IV), L-cysteine (200 nmol/kg/min, IV), or S-nitroso-D-cysteine (D-CSNO, 200 nmol/kg/min, IV). The data are shown as mean ± SEM. There were 8 rats in each group. *P < 0.05, significant change from baseline. †P < 0.05, L-cysteine or D-CSNO versus vehicle.

**Supplementary Figure S6**

**A.**

**B.**

**C.**

**D.**

**Supplementary Figure S6.** **Panels A and B:** Total arithmetic changes in non-eupneic breathing index (NEBI) and NEBI/Frequency over the nine-minute period following the injection of naloxone methiodide (1.5 mg/kg, IV) in rats receiving intravenous infusions of vehicle (20 μL/min, IV) or S-nitroso-L-cysteine (L-CSNO) at 100 nmol/kg/min (L-CSNO 100), or 200 nmol/kg/min (L-CSNO 200). **Panels C and D:** Total arithmetic changes in NEBI and NEBI/Frequency over the nine-minute period following injection of naloxone methiodide (1.5 mg/kg, IV) in rats receiving intravenous infusions of vehicle (20 μL/min, IV), L-cysteine (L-CYS, 200 nmol/kg/min, IV) or S-nitroso-D-cysteine (D-CSNO, 200 nmol/kg/min, IV) The data are shown as mean ± SEM. There were 8 rats in each group. *P < 0.05, significant change from Pre values. †P < 0.05, L-CSNO 100 or L-CSNO 200 versus vehicle. †P < 0.05, L-CYS 200 or D-CSNO 200 versus vehicle.

**Supplementary Table S6**

Tail-flick latencies at various timepoints for five treatment groups

|  |  |  | **Tail-flick latencies (sec) in the treatment groups** | | | | | | | | |
| --- | --- | --- | --- | --- | --- | --- | --- | --- | --- | --- | --- |
| **Fentanyl** | **Phase** |  | **Vehicle 1** |  | **Vehicle 2** |  | **L-CSNO** |  | **L-Cysteine** |  | **D-CSNO** |
|  | Baseline |  | 2.5 ± 0.1 |  | 2.5 ± 0.1 |  | 2.6 ± 0.1 |  | 2.4 ± 0.1 |  | 2.4 ± 0.1 |
|  | Infusion |  | 2.5 ± 0.1 |  | 2.6 ± 0.1 |  | 2.5 ± 0.1 |  | 2.5 ± 0.1 |  | 2.4 ± 0.1 |
| 10 μg/kg | 15 min |  | 2.5 ± 0.1 |  | 11.6 ± 0.2 |  | 12.0 ± 0.0 |  | 11.4 ± 0.2 |  | 11.7 ± 0.2 |
|  | 30 min |  | 2.4 ± 0.1 |  | 8.2 ± 0.5 |  | 11.4 ± 0.2 |  | 8.5 ± 0.6 |  | 8.6 ± 0.4 |
| 25 μg/kg | 15 min |  | 2.5 ± 0.1 |  | 11.9 ± 0.1 |  | 12.0 ± 0.0 |  | 11.9 ± 0.1 |  | 11.8 ± 0.1 |
|  | 30 min |  | 2.6 ± 0.1 |  | 9.6 ± 0.6 |  | 11.9 ± 0.1 |  | 9.5 ± 0.5 |  | 9.3 ± 0.5 |
| 50 μg/kg | 15 min |  | 2.4 ± 0.1 |  | 12.0 ± 0.0 |  | 12.0 ± 0.0 |  | 12.0 ± 0.0 |  | 12.0 ± 0.0 |
|  | 30 min |  | 2.5 ± 0.1 |  | 11.3 ± 0.3 |  | 12.0 ± 0.0 |  | 11.2 ± 0.2 |  | 11.4 ± 0.3 |
| Drug | 5 min |  | 2.5 ± 0.1 |  | 8.6 ± 0.4 |  | 10.0 ± 0.3 |  | 8.5 ± 0.3 |  | 8.4 ± 0.3 |
|  | 15 min |  | 2.6 ± 0.1 |  | 7.9 ± 0.4 |  | 9.2 ± 0.3 |  | 7.8 ± 0.2 |  | 8.0 ± 0.3 |

L-CSNO, S-nitroso-L-cysteine. D-CSNO, S-nitroso-D-cysteine. Baseline denotes values prior to commencing the infusions. Infusion denotes the value immediately preceding injection of the first dose of fentanyl (10 μg/kg, IV). The group denoted Vehicle 1 received infusion of vehicle, 3 intravenous injections of vehicle, instead of the 10, 25 or 50 μg/kg doses of fentanyl, and 30 min after the third IV injection of vehicle, an injection of naloxone methiodide (2.5 mg/kg, IV). The rows designated Drug present values after injection of vehicle (Vehicle 2) or for all other groups, naloxone methiodide (2.5 mg/kg, IV). Data are shown as mean ± SEM. There were 6 rats in each group. The baseline values of each group were similar to one another (P > 0.05, for all comparisons). The infusion values immediately preceding injection of the first dose of fentanyl (10 μg/kg), were also similar to one another (P > 0.05, for all comparisons based on arithmetic changes).

**Supplementary Table S7**

Arithmetic changes in tail-flick latencies from those at 60 min of infusion compared to various timepoints in all treatment groups

|  |  |  | **Tail-flick latencies (sec) in the treatment groups** | | | | | | | | |
| --- | --- | --- | --- | --- | --- | --- | --- | --- | --- | --- | --- |
| **Fentanyl** | **Phase** |  | **Vehicle 1** |  | **Vehicle 2** |  | **L-CSNO** |  | **L-Cysteine** |  | **D-CSNO** |
| 10 μg/kg | 15 min |  | +0.1 ± 0.1 |  | +9.1 ± 0.2* |  | +9.5 ± 0.1* |  | +8.9 ± 0.1* |  | +9.3 ± 0.2* |
|  | 30 min |  | 0.0 ± 0.1 |  | +5.6 ± 0.4* |  | +9.0 ± 0.2* |  | +6.1 ± 0.6* |  | +6.2 ± 0.4* |
| 25 μg/kg | 15 min |  | +0.1 ± 0.1 |  | +9.4 ± 0.1* |  | +9.5 ± 0.1* |  | +9.4 ± 0.2* |  | +9.4 ± 0.2* |
|  | 30 min |  | +0.2 ± 0.1 |  | +7.1 ± 0.5* |  | +9.4 ± 0.1* |  | +7.0 ± 0.5* |  | +6.9 ± 0.5* |
| 50 μg/kg | 15 min |  | 0.0 ± 0.1 |  | +9.5 ± 0.1* |  | +9.5 ± 0.1* |  | +9.6 ± 0.1 |  | +9.6 ± 0.1* |
|  | 30 min |  | 0.0 ± 0.1 |  | +8.7 ± 0.3* |  | +9.5 ± 0.1* |  | +8.7 ± 0.2* |  | +8.9 ± 0.3* |
| Drug | 5 min |  | 0.0 ± 0.1 |  | +6.1 ± 0.4* |  | +7.5 ± 0.3*^,†^ |  | +6.1 ± 0.3* |  | +6.0 ± 0.4* |
|  | 15 min |  | +0.1 ± 0.1 |  | +5.3 ± 0.5* |  | +6.7 ± 0.4* |  | +5.3 ± 0.2* |  | 5.5 ± 0.3* |

L-CSNO, S-nitroso-L-cysteine. D-CSNO, S-nitroso-D-cysteine. The group denoted Vehicle 1 received an infusion of vehicle, 3 intravenous injections of vehicle, instead of the 10, 25 or 50 μg/kg doses of fentanyl, and 30 min after the third vehicle injection, an injection of naloxone methiodide (2.5 mg/kg, IV). The rows designated Drug present values after injection of vehicle (Vehicle 2) or for all other groups, naloxone methiodide (2.5 mg/kg, IV). Data are shown as mean ± SEM. There were 6 rats in each group. *P < 0.05, significant change from infusion values shown in Supplementary Table S6. ^†^P < 0.05, L-CSNO 100 or L-CSNO 200 versus Vehicle 2.
